# Insights into the evolution and regulation of miRNAs from the view of their DNA replication temporal domains

**DOI:** 10.3389/fgene.2025.1544802

**Published:** 2025-06-23

**Authors:** Xudong Wu, Tingting Liu

**Affiliations:** School of Engineering, Guangzhou College of Technology and Business, Guangzhou, China

**Keywords:** miRNA, replication temporal domains, hairpin structure, dicer cleavage site, miRNA promoters, predictive model

## Abstract

**Introduction:**

The DNA replication of eukaryotes proceeds in a defined temporal sequence known as the replication timing (RT) program. A recent study revealed that the early- and late-replication temporal domains have different DNA mutation patterns and that the late-replicating sequences have a substitution pattern biased towards A and T. It raises the interesting question of how the miRNAs in the late-replication domain cope with the mutation bias caused by RT.

**Methods:**

In this study, we characterized the genomic distribution of pre-miRNAs in relation to DNA replication timing, and identified 362 pre-miRNAs within late-replicating domains (late-miRNAs) and 631 pre-miRNAs within early-replicating domains (early-miRNAs). We comprehensively examined the multiple molecular features including the secondary structural properties, the genomic sequences surrounding the pre-miRNA loci, the Dicer processing motifs, and CAGE tag-based promoters and miRNAs expression profiles. Furthermore, we performed the simulation of miRNA-target regulatory networks to elucidate the co-regulation patterns among late-miRNAs. To advance predictive capabilities, we developed a a support vector machine (SVM) classifier based on RNA-FM embedding, enabling prediction of miRNAs’ replication timing domains.

**Results and Discussion:**

Our study indicated that the late pre-miRNAs maintained their ability to fold into hairpin structures through extending their lengths at both ends under the premise of maintaining a certain GC content of the precursors. The simulation demonstrated that the late-miRNAs tend to synergistically regulate the same genes and are involved in small molecule metabolism, immune responses and so on. The comparative analysis of early- and late- miRNAs confirmed that the information of replication timing domains is inherently encoded in miRNAs’ sequence-structure signatures, and suggested that late-replication specific mutation patterns leave direct imprints on miRNA architecture. This study provides insights into the impact of DNA replication timing on miRNA-mediated posttranscriptional regulation and helps us understand the evolutionary mechanism of miRNAs.

## Introduction

MicroRNAs (miRNAs) are important regulatory factors that direct the posttranscriptional regulation of target genes. The hairpin structure of the pri-miRNA transcript is recognized and cleaved by Drosha, and the 80-120 nt pre-miRNA is translocated from the nucleus to the cytoplasm by RanGTP/exportin 5 (Ambros, 2011; Ambros and Ruvkun, 2018). The stem of the hairpin structure is then recognized and cleaved by Dicer, releasing the mature miRNA molecule with 18–20 bp in length. In this way, the pre-miRNA encodes an 18–25 bp mature miRNA at the 5′end or 3′end; if both have high stability in the cytoplasm, this pre-miRNA is considered to encode both the5′ miRNA and 3′miRNA.

In mammals, the 5′seven- or eight-bp sequence of mature miRNA recognizes the target gene by binding to the 3′UTR of the reverse-complementary mRNA. In general, for target genes with high expression intensity, miRNAs may reduce transcriptional noise by hindering protein translation (Pasquinelli, 2012). For target genes whose expression is low, miRNAs may directly regulate mRNA abundance by promoting mRNA degradation (Wu and Song, 2011; Wu et al., 2012).

The DNA replication of eukaryotes proceeds in a defined temporal sequence known as the replication timing (RT) program. RT is dynamically regulated during development and exhibits cell type-specific RT signatures, which are closely related to the A/B compartments of the chromatin structure, the local chromatin environment and the transcriptional potential (Rivera-Mulia and Gilbert, 2016). In mammals and other warm-blooded vertebrates, the timing of DNA replication is associated with long DNA segments with uniform GC base compositions, known as GC isochores, which are differentially enriched between early and late replication domains (Vinogradov, 2003; Costantini and Bernardi, 2008). The GC isochores attributed by the RT of germ cells,can be fixed during evolution. Late-replicating sequences have a substitution pattern biased towards A and T, attributed to the deamination of methylcytosine at CpG sites and their intensity. Therefore, in germline cells, the initiation timing of replication leads to a preference for DNA mutation transitions (C- > T and G- > A) in late-replicating domains during evolution (Chen et al., 2010; Cornejo-Páramo et al., 2024).

This raises the intriguing question of how miRNAs in the late stage of replication cope with the mutation bias caused by RT. In terms of the miRNA maturation mechanism, pri-miRNAs must encode stable hairpin structures with low folding free energy to be recognized by Drosha in the nucleus (Rice et al., 2020; Roden et al., 2017). After pruning, they are recognized by ExportinS for nuclear export. Finally, the stem region of the hairpin structure is cleaved by Dicer to release mature miRNAs (Zapletal et al., 2023). In this study, we explored the evolutionary mechanism of miRNAs in replication domains with different mutation patterns, and performed a comparative analysis of early replication miRNAs (early-miRNAs) and late replication miRNAs (late-miRNAs) in terms of secondary structural properties, evolutionary depth, expression profiles, and the biological functions of target genes.

## Materials and methods

### Identification of early-miRNAs and late-miRNAs

A previous study investigated the DNA replication patterns of mouse primordial germ cells (PGCs) and spermatogonial stem cells (SSCs) through oligonucleotide hybridization microarray technology (Yehuda et al., 2018). Briefly, the sequencing reads were mapped against the mouse genome (mm9), the G1 phase reads were binned to establish genomic windows, and S phase reads were counted to determine the S/G1 ratio. The ratio data was normalized by subtracting the mean and dividing by the standard deviation. In each cell line, the genomic windows were recorded as early domains if ratio>0, and the genomic windows were recorded as late domains if ratio ≤ 0.

The chromosomal coordinates of *Mus musculus* pre-miRNAs (GRCm38/mm10) were downloaded from the miRBase (v22) (Kozomara et al., 2019). The genomic coordinates were converted from mm10 to mm9 through the LiftOver tool and then intersected with the regions of the replication domains through the bedtools tool (Quinlan and Hall, 2010). A total of 993 pre-miRNAs were found to fall within the regions with early and late replication signals available. The 362 pre-miRNAs located in DNA in late replicating domains in at least one sample were defined as late-miRNAs, and the remaining 631 pre-miRNAs that were consistently located in early replicating domains were defined as early-miRNAs.

### Secondary structural analysis of miRNA hairpins

The sequences of pre-miRNAs were downloaded from the miRBase (v22), and the frequencies of mononucleotide and dinucleotide sequences were calculated separately. According to the identifiers of pre-miRNAs, the minimum free energy structures were downloaded from the miRBase (v22). Through forgi software (Varenyk et al., 2023), the counts of nucleotides involved in perfect matches, mismatches, bulges, and loops were calculated. Through seqfold software (Ouya et al., 2013), the decomposed terms of the folding free energy, including the free energy of matches, mismatches, bulges, and loops, were calculated. Through RNAfold software (Varenyk et al., 2023), the energy of the minimum free energy structure, mef_freq (frequency of the mfe structure in the ensemble), and diversity (ensemble diversity) were calculated. The neutral set sizes of the miRNA structures were determined via the method described by Martin and Ahnert, (2022).

### Genomic sequence analysis of pre-miRNA loci

The coordinate information of the mouse-rat genome alignment was downloaded from UCSC (Mm9 vs. Rn5 genome alignment) (Zhao and Zhang, 2015). Through LiftOver, the orthologous regions of mouse miRNAs in the rat genome were determined. The rat genome sequence was downloaded from UCSC (Rn5), and the sequences of the miRNA orthologues were extracted. Using the global alignment algorithm of ClustalW (Larkin et al., 2007), the mouse miRNAs were aligned against the rat orthologous sequences, and the sequence similarity was calculated via the distmat in the EMBOSS toolbox (Rice et al., 2000). Finally, the homologous sequences with a similarity ≥ 60% were retained. The minimum free energy secondary structure was determined by RNAfold, and the ensemble parameters of the filtered sequences were obtained.

The genome sequence of the mouse was downloaded from UCSC (mm9). On the basis of the genomic coordinates of pre-miRNA, the sequences 200 bp upstream, 50 bp from the 5′end of pre-miRNA, 50 bp before the 3′end of pre-miRNA, and 200 bp downstream were extracted. To calculate the site-based GC ratio, the nucleotides of G or C were counted for each site and divided by the number of early-miRNAs and late-miRNAs, and the GC content profiles were obtained for early-miRNAs and late-miRNAs, respectively. A T test was used to investigate whether there was a significant difference in the GC ratios between the 200 bp upstream sequence and the 5′50 bp sequence of the miRNAs and whether there was a significant difference in the GC ratios between the 200 bp downstream sequence and the 3′50 bp sequence of the pre-miRNAs.

On the basis of the genomic coordinates of pre-miRNAs, 10 kb upstream and downstream of the miRNAs and 1 Mb (1,000 kb) were identified. The general feature files of the mouse protein-coding genes, including the encoding coordinates of the genes, exons and introns, were downloaded from UCSC (mm9), and the overlap analysis was performed via the intersect tool of the bedtools package. For each miRNA, whether there was a protein-coding gene within its 10 kb range was recorded, and the number of protein-coding genes within the 1 Mb range was calculated. For miRNAs located within protein genes, the number of miRNAs overlapping with the same and opposite strand was calculated respectively.

### The analysis of the dicer processing motifs

The positional information of mature miRNAs mapped to precursor miRNAs (pre-miRNAs) were retrived from miRBase, and the Dicer cleavage patterns were characterized through the following analytical framework: the 5′cleavage recognition motif was defined as second to ninth nucleotides downstream of the 3′-end of 5′mature miRNAs, the 3′cleavage recognition motif was defined as −3rd to −10th nucleotides upstreamn of the 5′-end of 3′mature miRNAs (Liu et al., 2021). The position-specific nucleotide distributions of the Dicer 5′ cleavage and 3′ cleavage motifs were compared between the early- and late-miRNAs using Chi-square tests.

To perform the unsupervised motif clustering analysis, a three-stage computational pipeline was implemented as follows, (1) For the unique sequence motifs from the identified cleavage windows, the pairwise edit distances were calculated using levenshtein distance metric (minimum character insertions, deletions, or substitutions required for sequence alignment), and then the n × n distance matrix was constructed, where n equal to the counts of the unique motifs. (2) The dimensionality reduction was conducted using the multidimensional scaling (MDS) via sklearn.manifold.MDS with the n_components was set as 2, the high-dimensional distance matrix D was transformed into 2D embedding space. (3) The spatial separation between early- and late-replicating miRNA clusters in MDS projection space was evaluated through Kernel density estimation, and the two-sample t tests were performed along the first and the second principal dimension separately.

### Analysis of miRNA expression profiles

Second-generation sequencing-based transcriptome sequencing is a high-throughput and unbiased method for determining the expression profile of mature miRNAs. A previous study constructed mature miRNA libraries and generated transcriptomic profiles across 7 mouse tissues (Keller et al., 2022). The reads per million mapped reads (RMPs) of miRNAs were downloaded, and the expression intensities of biological replicates of each tissue were averaged, the ≥ 1 rpm was selected as the criterion to determine whether the miRNA was expressed in the tissue. The proportions of early-miRNAs and late-miRNAs expressed in each tissue were calculated.

### CAGE tag processing and the identification of miRNA promoters

In order to identify the miRNA promoters with high confidence, the analytical workflow for CAGE tag quantification and semi-supervised mixture modeling construction was implemented as follows (Marsico et al., 2013), (1) The mouse pre-mapped CAGE tags were retrieved from the FANTOM4 database (Kawaji et al., 2009), the genomic coordinates of CAGE tags were intersected with the 5′ upstream 50 kb region relative to each annotated pre-miRNA via the intersect tool of the bedtools package (Quinlan and Hall, 2010). The raw tag count matrix was created and the position-specific quantile normalization was applied to mitigate systematic inter-sample variations affecting absolute tag counts, ensuring comparable signal distributions across experimental replicates. (2) The abundance distribution of CAGE signals was modeled as a two-component mixture of promoter-associated regions and background noise: (a) For the background noise modeling, the 1,000 intergenic locus were randomly selected genome-wide, the CAGE tags within ±2,500 bp windows centered on these loci were extracted, and the tags detected in fewer than 5 samples were classified as background noise. (b) For selection of the candidate promoters, the tags within miRNA upstream regions (−50 kb from pre-miRNA) were designated as putative promoters, the 1,000 bp windows centered on each tag midpoint were extracted and the sequence properties were calculated, including CpG ratio, TATA-box affinity (position weight matrix scoring by the TRAP method) (Thomas-Chollier et al., 2011), and the average PhastCons scores. Based on these features, the binomial generalized linear models (GLMs) were constructed to discriminate the putative promoters vs. background. (3) A semi-supervised expectation-maximization (EM) framework was employed to model tag counts: (a) the precomputed promoter probabilities from GLM outputs were incorporated as bayesian priors, (b) the mean and standard deviation for each mixture component were learned by iteratively optimization via EM, (c) The posterior probability of each tags as background noise or putative promoter were calculated. (4) The tags were partitioned into promoter/background classes using a decision boundary of P(Promoter∣x) > 0.5.

The CAGE regions exhibiting both high prior probabilities and elevated tag densities were retained as validated promoters. For each miRNA, the tags with maximal tag density was designated as its high-confidence promoters.

### The construction of simulated miRNA‒target regulation networks

The experimentally verified miRNAtarget genes were downloaded from miRTarBase. The edge-swapping method was used to construct the simulated networks (Wu and Qi, 2010). A randomized network was constructed via the following steps: (a) Two miRNA‒target gene pairs, namely, miRNA1- > target1 and miRNA2- > target2, were randomly selected from the regulatory network. (b) If miRNA1- > target2 and miRNA2- > target1 do not exist in the network, edge swapping is performed; that is, the miRNAs are swapped between the two edges, miRNA1- > target1 is transformed to miRNA2- > target1, miRNA2- > target2 is transformed to transformto miRNA1- > target2, and the network is updated; otherwise, edge swapping is not performed. (c) Edge swapping from steps (a) to (b) was carried out 100,000 times. Eventually, there was≤10% of the miRNA‒target genes relationships overlapped between each of the randomized network and the original network.

According to the categories of miRNAs, the following values were counted: the number of target genes regulated exclusively by early-miRNAs, the number of target genes regulated exclusively by late-miRNAs, and the number of target genes jointly regulated by both early- and late-miRNAs. In the 100 randomly simulated networks constructed, the distributions of these three values were measured, and the significance of the deviation of these observed numbers from the random distribution was determined. The p-values were obtained from the Z-score, which was calculated as (the observed number - the mean number of 100 simulations)/standard deviation of 100 simulations.

To investigate whether the early- and late- DNA replication stages where miRNAs are located influence the early- and late- replication stages of their target genes, the following values were counted: the number of miRNA–target gene regulatory pairs within the same replication zone, including the number of regulatory relationships where early-miRNAs regulate early-target genes, the number of regulatory relationships where late-miRNAs regulate late-target genes; and the number of miRNA–target gene regulatory pairs across different replication zones, including the number of regulatory relationships where early-miRNAs regulate late-target genes and the number of regulatory relationships where late-miRNAs regulate early-target genes. In the constructed 100 random simulation networks, the distributions of these two values were measured, and the significance of the deviation of these observed characteristic numbers from the random distribution was determined. The p-values were obtained from the Z-score, which was calculated as (the observed number - the mean number of 100 simulations)/standard deviation of 100 simulations.

### Measurement of the expression variation of miRNA targets

miRNAs regulate the expression level of target genes at the posttranscriptional level and usually affect the stability of target mRNAs. The microarray expression profiles of 19 mouse tissues were downloaded from NCBI (GSE9954) (Thorrez et al., 2008). The expression intensity of each biological replicate was averaged across each tissue, and the tau value was used to measure the fluctuations in the expression of protein-coding genes across tissues (Yanai et al., 2005).

### Functional analysis of miRNA target genes

MSigDB (Molecular Signatures Database, Mouse collections) was used to collect gene annotations for functional enrichment analysis (Liberzon et al., 2011). The functional differences in the target genes of early-miRNAs and late-miRNAs were investigated at four levels: GO molecular function, GO biological process, GO cellular component, and KEGG metabolic pathway. In each annotation category, the numbers of target genes of early-miRNAs and late-miRNAs with annotations available were recorded. For each subclass, the proportions of target genes of early-miRNAs and late-miRNAs with annotations available were compared, and the significance of the differences was measured via the chi-square test.

### The construction of SVM model to predict the early- and late-miRNAs

In order to establish the predictive models for early- and late-miRNAs, the analytical workflow for miRNA features extraction and the svm classifier construction was implemented as follows, (1) the RNA-FM pre-trained model was employed for miRNA sequence feature learning (Shen et al., 2024): (a) The pre-trained RNA-FM model with a Transformer-based architecture and its corresponding vocabulary system (alphabet) were loaded using fm. pretrained.rna_fm_t12 (). (b) The miRNA sequences were converted into tensor formats via the batch_converter via esm library, generating batch data containing the encoded token tensors (batch_tokens) with dimensions as batch_size × sequence_length), (c) The processed data were fed into RNA-FM for forward propagation, extracting hidden states from the 12th layer with repr_layers=12 (dimensions: sequence_length × 640), then the column averaging along the sequence axis yielded a 640-dimensional feature vector per miRNA (dimensions: 1 × 640). (2) A support vector machine (SVM) was adopted to predict miRNA replication timing (early vs. late): (a) Data Partitioning & Hyperparameter Tuning: Feature matrices were split into training (80%) and test sets (20%) using train_test_split (sklearn.model_selection) with test_size = 0.2. (b) A hyperparameter grid was defined to optimize radial basis function (RBF) (kernel parameters: “C”: [0.1, 1, 10, 100, 1,000], “gamma”: [1, 0.1, 0.01, 0.001, 0.0001], “kernel”: [“rbf”], “probability”: [True]), this parameter space spanned four orders of magnitude for C and gamma, ensuring thorough exploration of model complexity and generalization trade-offs. (c) a class-weighted SVC was initialized, and the exhaustive grid search with 5-fold cross-validation was conducted to evaluate all hyperparameter combinations, with refit set as true to trigger automatic retraining of the optimal model on the full training set. The final model instantiation was executed via grid.fit. (3) The class membership probabilities were generated using grid.best_estimator_.predict_proba, where the predicted probability of late replication domain (approaching 1) or early domain (approaching 0) served as the score of late replication timing propensity. (4) The model performance was evaluated through the receiver operating characteristic (ROC) curve analysis with area under the curve (AUC) quantification, and the probability distributions between early- and late-replicating miRNA groups were compared by the non-parametric Mann-Whitney U tests.

## Results

### Comparison of secondary structural properties between early-miRNAs and late-miRNAs

**FIGURE 1:**
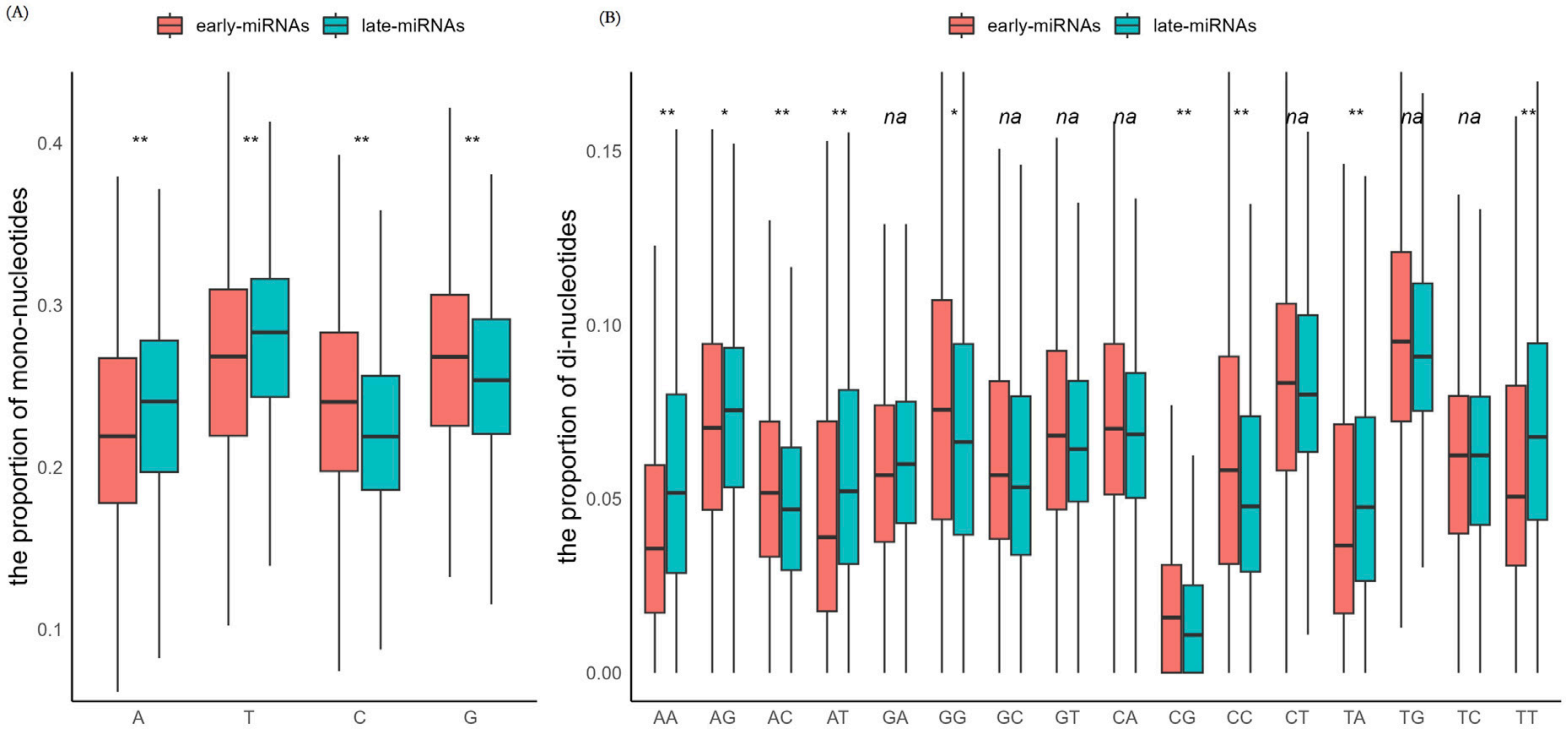
The early-miRNAs and late-miRNAs have significantly different sequence properties. **(A)** Comparison of the mononucleotide composition in the sequences of early- and late-pre-miRNAs. **(B)** Comparison of the dinucleotide compositions of the sequences of early- and late-miRNAs.The * represents a p value≤0.05, ** represents a p value ≤ 0.01, and na represents a p value > 0.05.

We investigated the distribution of mononucleotides and dinucleotides in the sequences of early pre-miRNAs and late pre-miRNAs (see *Methods*). In line with the biased accumulation of A or T in the late replicating domains, the late pre-miRNAs presented significantly greater proportionsof A and T, and significantly lower proportionsof G and C (Mann‒Whitney U test, *p* < 0.05, *two-sided*) (Figure 1A). In terms of the distribution of dinucleotide motifs, late pre-miRNAs presented significantly greater proportionsof AA, AG, AT, TA, and TT (Mann‒Whitney U test, *p* < 0.05, *two-sided*) and significantly lower proportionsof AC, GG, CG, and CC (Mann‒Whitney U test, p < 0.05, *two-sided*) (Figure 1B).

We investigated the energy characteristics of the secondary structure via three measures: the minimum free energy (mfe) structure, the frequency of the mfe structure in the ensemble and the ensemble diversity (see *Methods*). Early pre-miRNAs and late pre-miRNAs have similar distributions of mfe energy, mfe frequency and ensemble diversity (Mann‒Whitney U test, *p* > 0.05, *two-sided*) (Supplementary Figures S1A–C); that is, both early pre-miRNAs and late pre-miRNAs can fold into stable hairpin structures.

**FIGURE 2:**
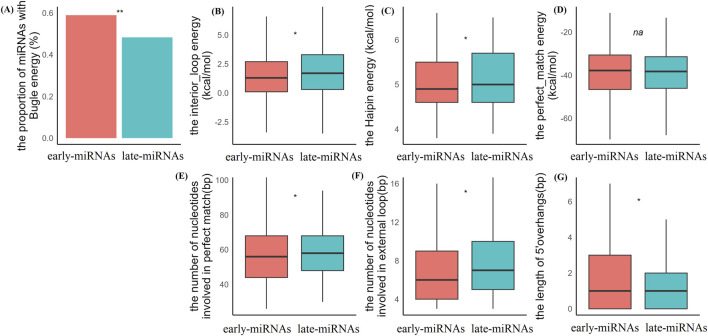
The early-miRNAs and late-miRNAs have significant structural properties. **(A)** Comparison of energy terms with bugle contributions in the minimum energy structure of early- and late-pre-miRNAs. **(B)** Comparison of interior loop energy in the minimum energy structure of early- and late-pre-miRNAs. **(C)** Comparison of haipin energy in the minimum energy structure of early- and late-pre-miRNAs. **(D)** Comparison of perfect match energy in the minimum energy structure of early-and late-pre-miRNAs. **(E)** Comparison of the length of perfect match nucleotides in the minimum energy structure of early-and late-miRNAs. **(F)** Comparison of the length of external loop nucleotides in the minimum energy structure of early- and late-miRNAs. **(G)** Comparison of the length of 5′overhangs in the minimum energy structure of early-and late-miRNAs.The * represents a p value≤0.05, ** represents a p value ≤ 0.01.

We analyzed the energy components contributing to the minimum free energy of the hairpin structures (see Methods). In a canonical hairpin structure, the insertions or deletions occurring in the stem region introduce bulges, which have destabilized effect on the structure and are reflected in the energy term BULGE. Fifty-nine percent of the early-replicated miRNAs had energy contributions from BULGE, whereas 48% of the late-replicated miRNAs had energy contributions from BULGE (*chi-square*test, *p* < 0.5, *df* = 1, *two-sided*) (Figure 2A). The point mutations in the middle of the stem also destabilized the structure and were reflected in the energy terms of STACK-mismatch (tri-nucleotide alignment with middle mismatch) and INTERIOR-loop. We found that late-miRNAs have significantly greater energy contributions from the INTERIOR-loop (Mann‒Whitney U test, *p* < 0.05, *two-sided*) (Figure 2B). The mutations occurring near the ends of the hairpin loops in the stem lead to loop enlargement and a typical disordered region, which destabilizes the secondary structure. Although the destabilizing effect of the loop on the secondary structure cannot be precisely characterized, the base pair near the disordered loop can provide a rough estimate and is reflected in the energy term HAIPIN. We found that late miRNAs have higher HAIPIN energy values (Mann‒Whitney U test, *p* < 0.05, two-sided) (Figure 2C), suggesting weaker restraint to the disordered loop. The perfect matches have a predominant contribution to structural stability, and as reflected in the energy term STACK-match, the late-miRNAs and early-replicated miRNAs have similar values of STACK-match energies (Mann‒Whitney U test, *p* > 0.05, *two-sided*) (Figure 2D).

From the perspective of binding energy, A:T basepairing consists of two covalent bonds, whereas G:C basepairing consists of three covalent bonds, and the binding energy of A:T basepairing is significantly lower than that of G:C basepairing. Given the same length, hairpin structures with high A/T contents generally have significantly higher free energies than hairpin structures with high C/G contents and are therefore less tolerant of destabilized mutations. To reduce the destabilization of mutation and loop enlargement, hairpin structures with high A/T contents can be stabilized by adopting the longer stem or extending the stem length. In line with this inference, although the late pre-miRNAs have longer loops, they have the significantly higher number of nucleotides involved in perfect matching (Mann‒Whitney U test, p < 0.05, *two-sided*) (Figures 2E, F). In line with the biased accumulation of A/T mutations in late replication domains, late-miRNAs may obtain matched A:T pairs at both ends, and we observed that late-miRNAs presented significantly shorter 5′end overhangs than early-miRNAs did (Mann‒Whitney U test, *p* < 0.05, *two-sided*) (Figure 2G).

### Comparison of evolutionary properties between early-miRNAs and late-miRNAs

From the perspective of the mechanisms of miRNA processing and maturation, miRNAs are predominantly determined by their ability to form stable hairpin structures. Therefore, the preservation of a stable secondary structure is key for the survival of nascent miRNAs during evolution. In the late stage of replication, with biased accumulation of A/T bases, miRNAs may be prone to obtain A:T base pairings and expand the length of alignments.

**FIGURE 3:**
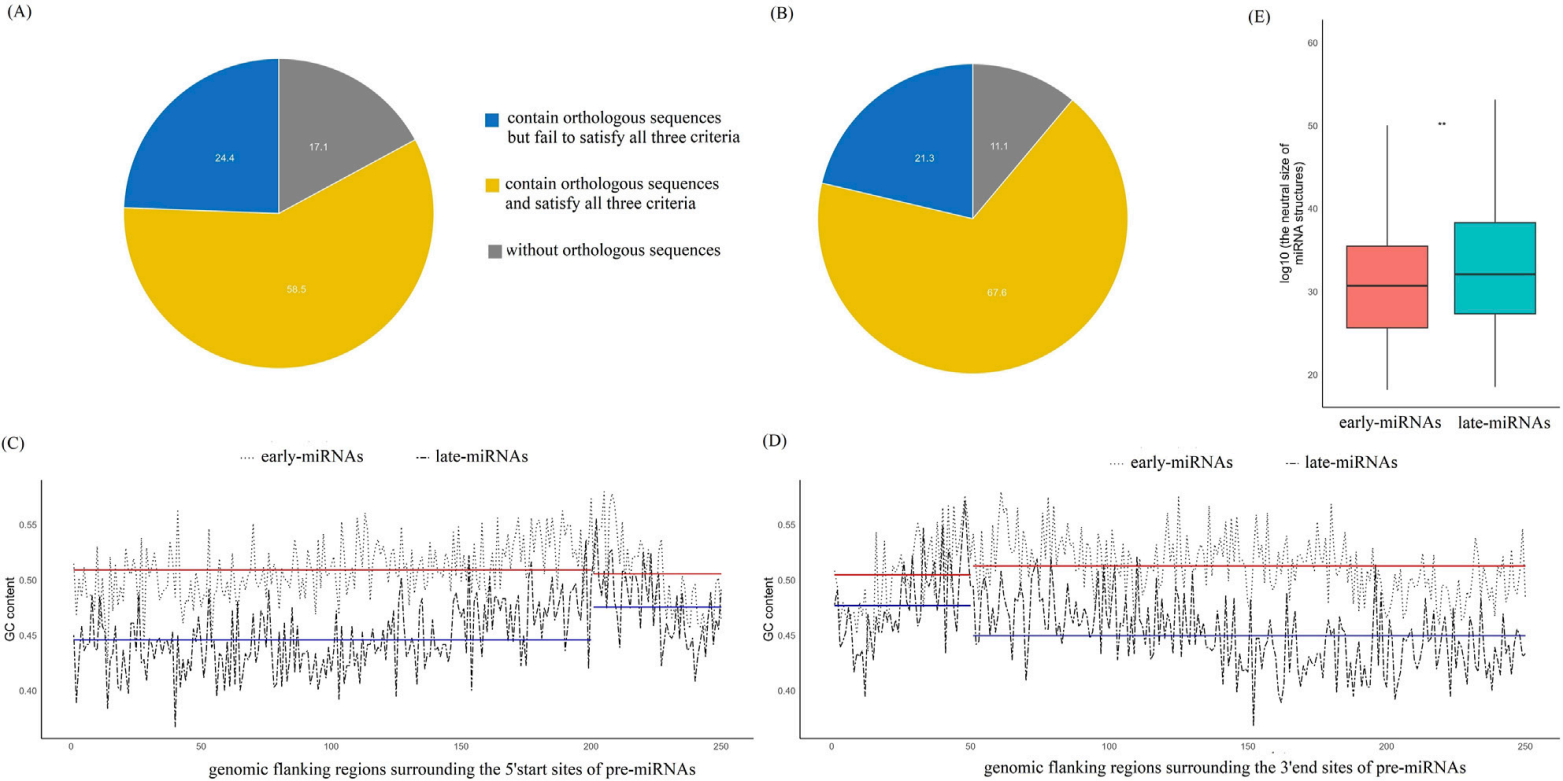
The early-miRNAs and late-miRNAs have significantly different evolutionary profiles and GC contents. **(A)** Distribution of the number of earlypre-miRNAs in three categories: pre-miRNAs without orthologues, pre-miRNAs with orthologous genes that meet with <three energy criteria, and pre-miRNAs with orthologous genes that meet the three energy criteria. **(B)** Distribution of the number of late pre-miRNAs in three categories: pre-miRNAs without orthologues, pre-miRNAs with orthologous genes that cannot meet with the three energy criteria, and pre-miRNAs with orthologous genes that meet with the three energy criteria. **(C)** GC content profile of the pre-miRNAs and their upstream sequences; the positions from 1 to 200 bp represent the 200 bp upstream sequence, and from 201 to 250 represents the 50 bp sequence from the 5′end of the miRNA. The red lines represent the average GC content of early-miRNAs and their upstream sequences. The bluelines represent the average GC content of early-miRNAs and their upstream sequences. **(D)** GC content profile of the pre-miRNAs and their downstream sequences; the positions from 1 to 50 bp representthe 50 bp sequence to the 3′end of the miRNA,and from 51 to 250 represent the 200 bp downstream sequence. The red lines represent the average GC content of early-miRNAs and their downstream sequences. The bluelines represent the average GC content of late-miRNAs and their upstream sequences. **(E)** Comparison of the size of the neutral set between the early- and late-miRNAs. The **represents a p value ≤ 0.01.

We investigated the orthology sequences of pre-miRNAs in the mouse-rat genomic alignments (see Methods). The proportion of late pre-miRNAs with homologous sequences in the rat genome was significantly greater than that of early-miRNAs (88.9% vs. 82.9%, *chi-square*test, *p* < 0.05, *df* = 1,*two-sided*) (Figures 3A,B). To investigate whether these orthologous regions encoded stable secondary structures, we calculated three energy measures of mouse miRNAs and obtained the 95th percentile of the folding energy of the minimum free-energy structure as −19.1 kcal/mol, the 95th percentile of the frequency of the mfe structure in the ensemble as 0.54, and the 95th percentile of the ensemble diversity as 22.2. We then extracted the rat orthologue sequence from the alignment and used *RNAFold* to investigate its folding potential. The orthologous sequences of rat satisfying the three criteria of dG <=−19.1 kcal/mol, mef_freq≤0.54 and ensemble_diversity ≤ 22.2 were selected as pre-miRNA candidates with high-quality folding potential. The results demonstrated that the homologous regions of late-miRNAs in the rat genome have a greater propensity to encode high-confidence pre-miRNA candidates (67.6% vs. 58.5%, *chi-square* test, *p* < 0.05, *df* = 1,*two-sided*) (Figures 3A,B). The late miRNAs benefit from the biased enrichment of A/T of the late-replicating domains and have a greater possibility of being retained in evolution.

We analysed the GC contents of the upstream and downstream sequences of the pre-miRNAs separately. Specifically, we extracted the upstream 200 bp sequence of the pre-miRNA and the first 50 bp sequence from the 5′end of the pre-miRNA. The GC content was calculated at each site from −200 to +50 bps, centering the 5′ end of the pre-miRNAs. The results revealed that there was no significant difference in the GC content between early miRNAs and their upstream sequences (T-test, *p* > 0.05, *two-sided*) (Figure 3C), whereas the GC content of the late-miRNAs was significantly greater than that of their upstream sequences (T-test, *p* < 0.05, *two-sided*) (Figure 3C). We extracted the last 50 bp sequence at the 3′end of the pre-miRNA and the 200 bp downstream sequence and calculated the GC content at each site from −50 to +200 bps, centering the 3′ end of the pre-miRNAs. The results revealed that there was no significant difference in the GC content between early miRNAs and their downstream sequences (T-test, *p* > 0.05, *two-sided*) (Figure 3D), the GC content of the 3′end sequences of late-miRNAs was significantly greater than that of their downstream sequences (T-test, *p* < 0.05, *two-sided*) (Figure 3D). These results suggest that early-miRNAs have similar nucleotide substitution patterns with upstream and downstream sequences, whereas late-miRNAs undergo purifying selection to maintain a certain CG content and pursue a stable hairpin structure under the pressure of domain-specific biased A/T-enriched mutations.

The genotype‒phenotype (GP) map of the RNA secondary structure connects RNA sequences with their corresponding secondary structures. For the evolution of the RNA secondary structure, the size of the neutral set of the structure, which quantifies how many sequences fold into this structure, has been proven to be particularly important. On the basis of the minimum energy free energy structure of miRNAs, we calculated the size of the neutral set of the structure of early-miRNAs and late-miRNAs (see Methods) and found that the size of the neutral set of late-miRNAs was significantly larger than that of early-miRNAs (Mann‒Whitney U test, p < 0.05, *two-sided*) (Figure 3E). This finding indicates that late miRNAs can accommodate more mutations while maintaining the folding potential.

### Comparison of transcription properties between early-miRNAs and late-miRNAs

**FIGURE 4:**
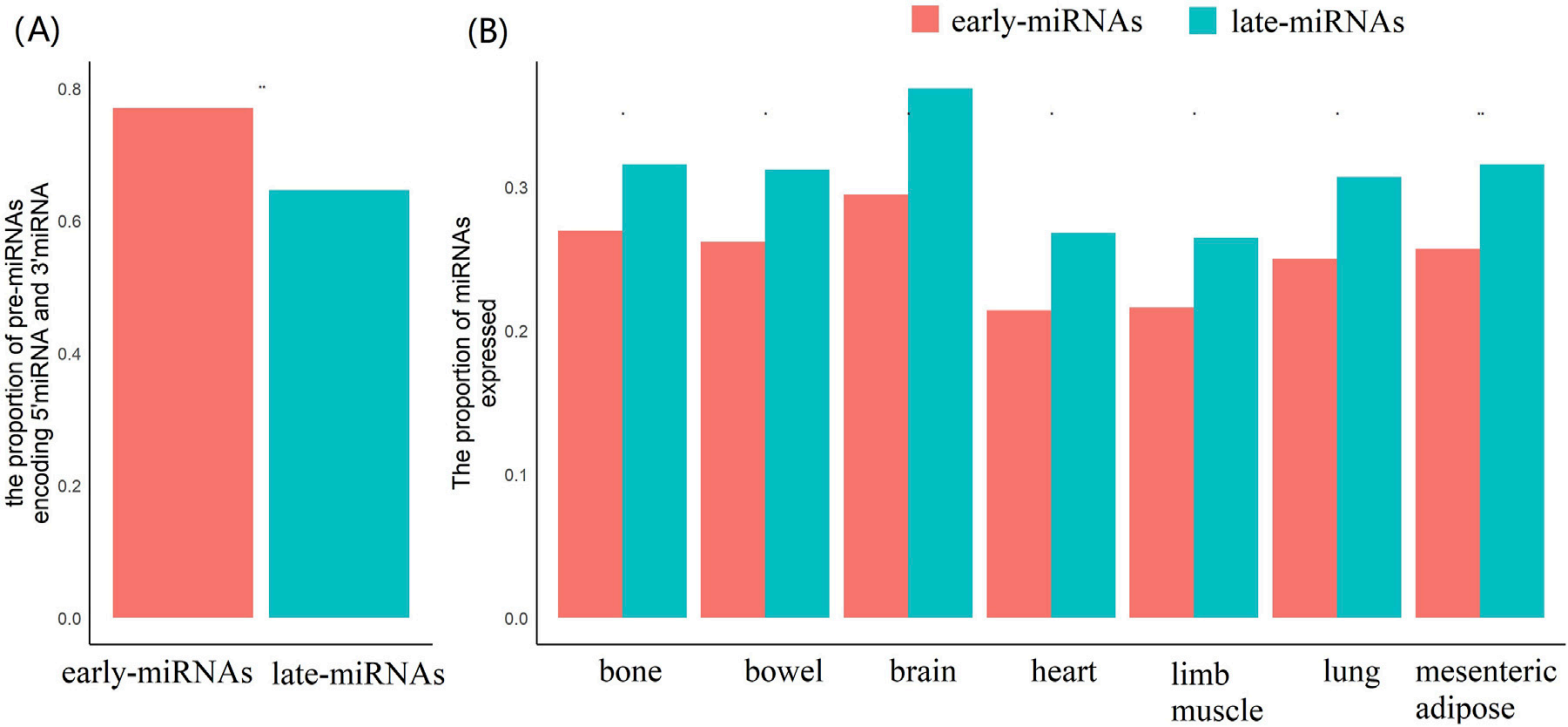
The early-miRNAs and late-miRNAs have significantly different encoding statues of mature miRNAs, and the expressional activities. **(A)** Comparison of the proportions of pre-miRNAs encoding both 5′end mature miRNAs and 3′end mature miRNAs. **(B)** Comparison of the expression activities of pre-miRNAs and late-miRNAs in the seven tissues. The * represents a p value≤0.05, ** represents a p value ≤ 0.01.

We examined mature miRNAs encoded by early pre-miRNAs and late pre-miRNAs. Among the 631 late pre-miRNAs, 486 pre-miRNAs encoded mature miRNAs at both the 5′and 3′ends. Among the 362 late pre-miRNAs, 234 pre-miRNAs encoded mature miRNAs at both the 5′and 3′ends. Apparently, the early pre-miRNAs tend to encode mature miRNAs at both the 5′and 3′ends (chi-square test, *p* < 0.05, *df* = 1,*two-sided*) (Figure 4A).

The maturation process of miRNAs depends on the precise cleavage of the secondary structure of pre-miRNAs by Dicer and the stability of miRNAs in the cytoplasm. On the basis of the positional coordinates of mature miRNAs in pre-miRNAs, we can identify sequence motifs around high-confidence Dicer cleavage sites.

Generally, the second to ninth nucleotides downstream of the 3′-end of mature 5′miRNAs embed its 5′high-confidence site, and −10th to −3rd nucleotides upstreamn of the 5′-end of mature 3′miRNAs embed its 3′high-confidence cleavage site. The results demonstrated late-miRNAs prefer to use A or U in the fifth positions in their 5′cleavage motifs (*Chi-square* test, *p* < 0.05, *df* = 1,*two-sided*) (Supplementary Figure S2A), and tend to adopt A or U in -8th, -6th, -3rd positions in their 3′cleavage motifs (*Chi-square* test, *p* < 0.05, *df* = 1,*two-sided*) (Supplementary Figure S2B), the cluster analysis of 5′cleavage motifs showed that the late-miRNAs have significant different values with the early-miRNAs in the first dimension in DMS projection (Supplementary Figure S3), and the cluster analysis of 3′cleavage motifs showed that the late-miRNAs have significant different values with early-miRNAs in both of the first and the second dimension in DMS projection (Supplementary Figure S4).

We collected the expression profiles of miRNAs in seven tissues and identified the expressed miRNAs in each tissue using a cut-off of rpm≥1 (see Methods). The results revealed significantly greater proportions of expressed late-miRNAs in each of the seven tissues (Mann‒Whitney U test, *p* < 0.05, *two-sided*) (Figure 4B).

We further used a probabilistic mixture model integrating the sequence based GLM priors and CAGE tags through a semi-supervised EM algorithm (see *Methods*), and identified 435 high-confidence promoters for 510 early-pre-miRNAs, and 174 high-confidence promoters for 246 late-pre-miRNAs (in both types of miRNAs, the clustered miRNAs were assigned with the same promoters). Comparative analysis of promoters properties revealed that the promoters of the late-miRNA exhibit significantly higher TATA-box propensity (Mann‒Whitney U test, *p* < 0.05, *two-sided*), the reduced CpG content (Mann-Whitney U test, *p* < 0.05, *two-sided*), and greater genomic distances from pre-miRNAs (Mann‒Whitney U test, *p* < 0.05, *two-sided*) (Supplementary Figures S5A-C). These promoter sequence divergences may suggest differential regulation by transcription factors with contrasting binding preferences (e.g., CG-rich versus AT-rich core motifs), and is involved in distinct cis-regulatory architectures for temporally regulated genes.

**FIGURE 5:**
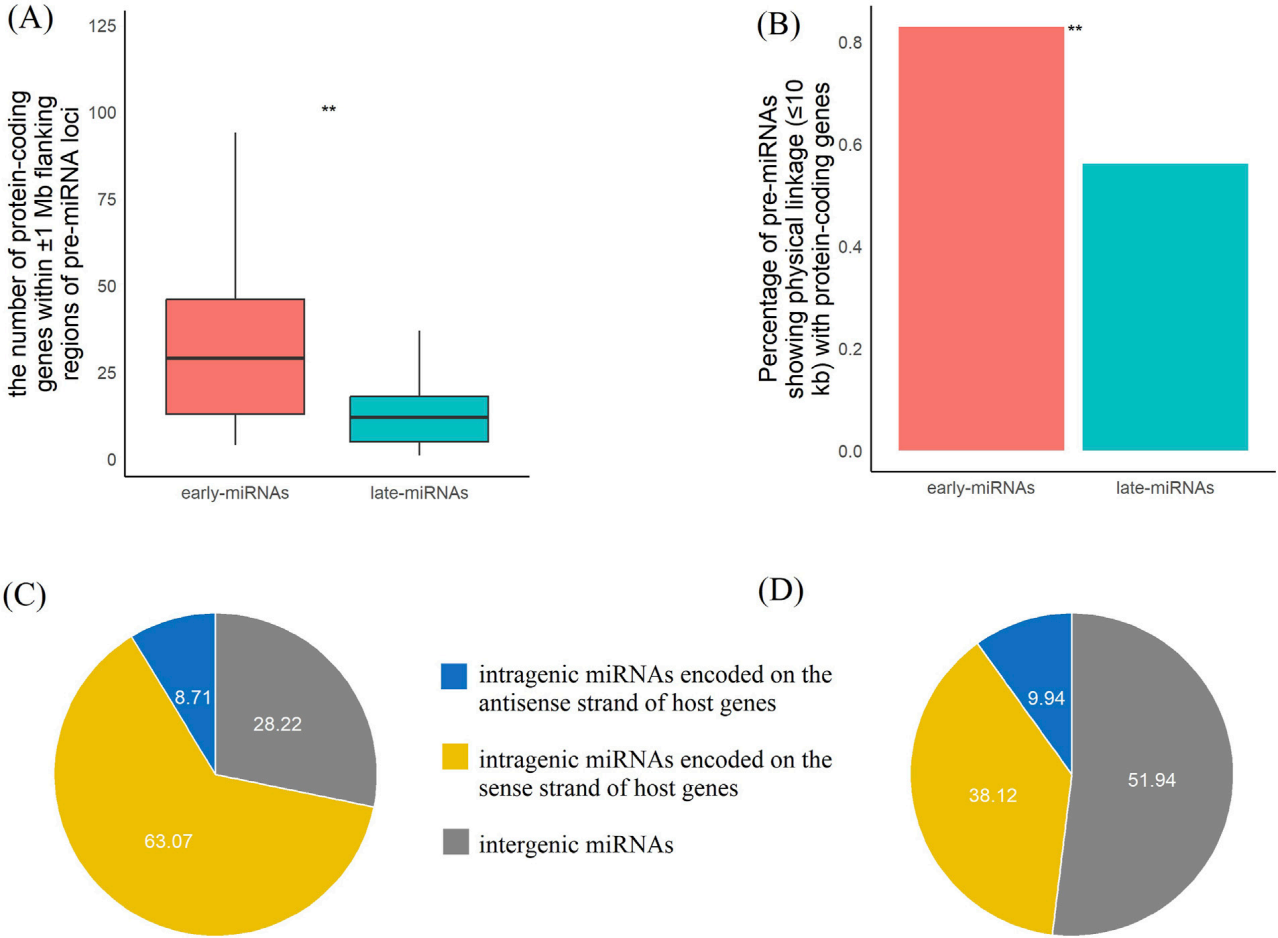
The early-miRNAs and late-miRNAs loci have significantly different transcription activities of protein-coding genes. **(A)** Comparison of the number of protein-coding genes within a 1 M range between early- and late-pre-miRNAs. **(B)** Comparison of the proportions of pre-miRNAs with protein-coding genes within the 10 kb range between early- and late-pre-miRNAs. **(C)** Distribution of the number of earlypre-miRNAs in three categories: pre-miRNAs with no intersection with protein-coding genes, pre-miRNAs located in protein-coding genes with the same strand, and pre-miRNAs located in protein-coding genes with the opposite strand. **(D)** Distribution of the number of late pre-miRNAs in three categories: pre-miRNAs with no intersection with protein-coding genes, pre-miRNAs located in protein-coding genes with the same strand, and pre-miRNAs located in protein-coding genes with the opposite strand. * represents a p value≤0.05, ** represents a p value ≤ 0.01.

Because miRNAs are involved in the posttranscriptional regulation of protein-coding genes, we further analyzed the overall transcriptional activity of the regions surrounding the miRNAs. There were significantly fewer genes surrounding ±1 M of the late-miRNAs than early-miRNAs (Mann‒Whitney U test, *p* < 0.05, *two-sided*) (Figure 5A). A total of 56% of the late miRNAs had protein-coding genes within the 10 kb range, which was significantly lower than the 83% of the early miRNAs (chi-square test, *p* < 0.05, *df* = 1,*two-sided*) (Figure 5B). We further analysed the overlap between pre-miRNAs and protein-coding genes. The 178 early pre-miRNAs did not overlap with protein-coding genes, whereas the 398 and 55 early pre-miRNAs overlapped with the same and opposite stand of host protien-coding transcripts, respectively. The 188 late pre-miRNAs did not overlap with protein-coding genes, whereas the 138 and 36 late pre-miRNAs overlapped the same and opposite stand of host protien-coding transcripts, respectively. The results demonstrated that early pre-miRNAs tend to be located in protein-coding regions (*chi-square* test, *p* < 0.05, *df* = 1,*two-sided*) (Figures 5C,D) and transcribe in the same direction as the transcripts of host protein-coding genes (*chi-square* test, *p* < 0.05, *df* = 1,*two-sided*) (Figures 5C,D).

These findings indicate that late miRNAs tend to be located in regions with lower protein-coding activity, may have relatively independent transcriptional initiation mechanisms, and are less likely to be affected by transcriptional noise. This may provide a beneficial effect for better exhibiting the role of posttranscriptional regulation.

### Late-miRNAs tend to synergistically regulate the same target genes

We investigated the topological properties of the posttranscriptional regulatory network mediated by early-miRNAs and late-miRNAs. On the basis of the experimentally validated target genes, we reconstructed a regulatory network, including 36,505 regulatory edges between 809 miRNAs (including 486 early-replicating miRNAs and 323 late-replicating miRNAs) and 7,411 target genes. We counted the number of target genes regulated exclusively by one early-miRNA, the number of target genes regulated exclusively by≥2 late-miRNAs, and the number of target genes regulated by both early-miRNAs and late-miRNAs. We adopted an edge-rewiring algorithm to construct a simulated regulatory network in which the number of regulatory miRNAs for each gene were the same as those in the original network (see *Methods*). We then constructed the number distribution of the genes regulated by each category of miRNAs.

**FIGURE 6:**
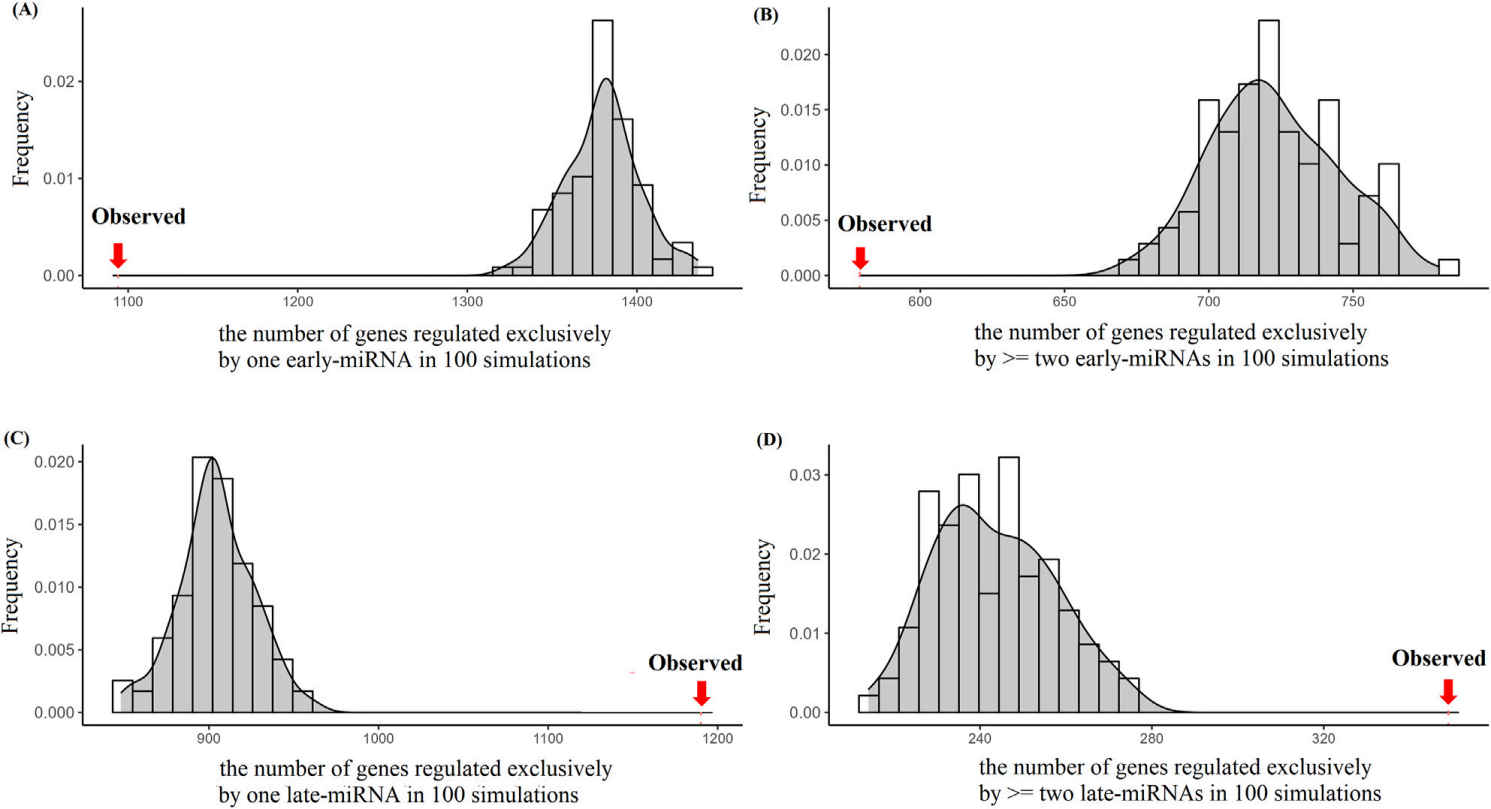
Simulation analysis to explore the target-recognition preference of late-miRNAs. **(A)** Distribution of the number of genes regulated exclusively by one early-miRNA in 100 simulations. The arrow on the left hand side represents the observed number of targets regulated exclusively by one early-miRNA. **(B)** Distribution of the number of genes regulated exclusively by≥two early-miRNAs in 100 simulations. The arrow on the left hand side represents the observed number of targets regulated exclusively by≥two early-miRNAs. **(C)** Distribution of the number of genes regulated exclusively by one late-miRNA in 100 simulations. The arrow on the right hand side represents the observed number of targets of one early-miRNAs. **(D)** Distribution of the number of genes regulated exclusively≥two late-miRNAs in 100 simulations. The arrow on the right hand side represents the observed number of targets regulated exclusively by≥two late-miRNAs.

We observed that 1,094 genes were regulated exclusively by one early-miRNA, and this value significantly deviated from the distribution observed in the simulated networks (1,379 ± 21.95, Z score = −13, *p* < 0.05) (Figure 6A). The 579 genes were regulated exclusively by ≥ 2 early-miRNAs, and this value significantly deviated from the distribution observed in the simulated networks (722 ± 21.99, Z score = -6.5, *p* < 0.05) (Figure 6B). The 1,190 genes were regulated by only one late miRNA, and this value significantly deviated from the distribution observed in the simulated networks (904 ± 21.95, Z score = 13.0, *p* < 0.05) (Figure 6C). The 349 genes were regulated by ≥ 2 late-replicating miRNAs, and this value significantly deviated from the distribution observed in the simulated networks (234 ± 13.85, Z score = 7.61, *p < 0.05*) (Figure 6D). The 4,199 genes were regulated by both early-miRNAs and late-miRNAs, and this value did not deviate from the distribution observed in the simulated networks (4,161 ± 23.34, Z score = 1.63, *p* > 0.05) (Supplementary Figure S6). Therefore, late-miRNAs tend to regulate more genes and tend to cooperatively regulate the same type of target genes.

We investigated whether the miRNA regulatory network has domain-like properties, that is, whether miRNAs and their target genes belong to the same replication temporal domain or different replication temporal domains. We further limited the analysis to target genes with early- or late-replication domain information. We observed 14,728 regulatory relationships of miRNA target genes in the same replication domain, including the number of early-genes regulated by early-miRNAs and the number of late-genes regulated by late miRNAs. This value does not significantly deviate from the distribution observed in the simulated networks (14,818 ± 73.44, Z score = −1.23, *p valu*e > 0.05) (Supplementary Figure S7A). We observed 13,215 regulatory relationships of miRNA target genes in different replication periods, including the number of early-genes regulated by late-miRNAs and the number of late-genes regulated by early-miRNAs. This value does not significantly deviate from the distribution observed in the simulated networks (13,124 ± 73.44, Z score = 1.23, *p value* > 0.05) (Supplementary Figure S7B). These results indicate that the target selection of miRNAs is independent of the replication temporal stages of the target genes.

We further analyzed the target genes regulated exclusively by late-miRNAs and the target genes regulated exclusively by early-miRNAs. The target genes regulated exclusively by late-miRNAs presented significantly shorter UTRs (Mann‒Whitney U test, *p* < 0.05, *two-sided*) (Supplementary Figure S8A) and lower expression variability (Mann‒Whitney U test, *p* < 0.05, *two-sided*) (Supplementary Figure S8B). These results indicate that late- and early-miRNAs indeed have certain effects on the regulation of target genes.

### Early-miRNAs and late-miRNAs involved in different biological functions

We investigated the biological functions of genes regulated exclusively by early-miRNAs (early miRNA targets) and those regulated exclusively by late-miRNAs (late miRNA targets) within cells. On the basis of the MSigDB annotation system (see *Methods*) (Liberzon et al., 2011), we conducted functional enrichment analyses in four categories: GO Molecular Function, GO Biological Process, GO Cellular Component, and KEGG metabolic pathways.

**FIGURE 7:**
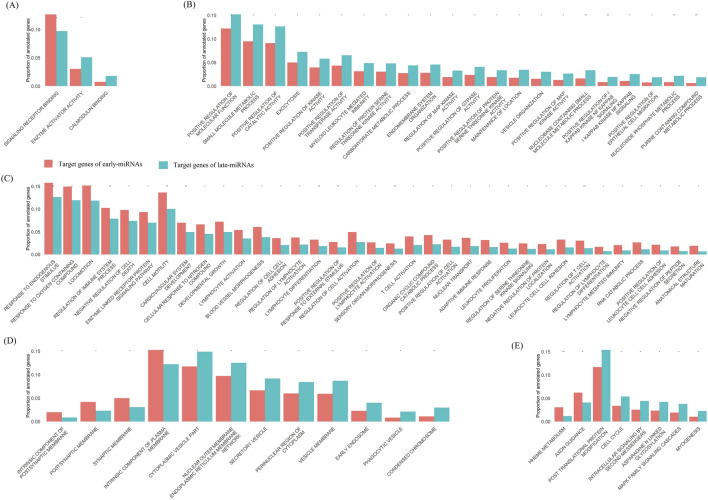
The target genes of early-miRNAs and late-miRNAs enriched in different biological function categories. **(A)** Comparison of the proportion of genes annotated by GO molecular functions of target genes between early-miRNAs and late-miRNAs. **(B)** Comparison of the proportion of genes annotated by GO biological processes of target genes between early-miRNAs and late-miRNAs, showing the GO terms associated with late-miRNA target enrichment. **(C)** Comparison of the proportion of genes annotated by GO biological processes of target genes between early-miRNAs and late-miRNAs, showing the GO terms associated with early-miRNA target enrichment. **(D)** Comparison of the proportion of genes annotated by GO cellular components of target genes between early-miRNAs and late-miRNAs. **(E)** Comparison of the proportion of genes annotated by KEGG metabolic pathways of target genes between early-miRNAs and late-miRNAs. * represents a p value≤0.05, and ** represents a p value ≤ 0.01.

We identified 1,224 early-miRNA target genes and 1,183 late-miRNA target genes with available annotations of GO molecular functions. Compared with the early-miRNA genes, the late-miRNA targets represented a significantly greater proportion of the genes annotated with enzyme activator activity and calmodulin binding (*chi-square* test, *p* < 0.05, *df* = 1, *two-sided*) and a significantly lower proportion of the genes annotated with signalling receptor binding (*chi-square* test, *p* < 0.05, *df* = 1, *two-sided*) (Figure 7A).

We identified 1,319 early-miRNA target genes and 1,259 late-miRNA target genes with available annotations of the GO Biological Process. Compared with the early-miRNA target genes, a significantly greater proportion of the late-miRNA target genes were annotated as exocytosis, regulation of protein serine and threonine kinase activity, regulation of MAP kinase activity, positive regulation of GTPase activity, etc. (*Chi-square* test, *p* < 0.05, *df* = 1,two-sided) (Figure 7B), and a significantly lower proportion of the target genes were annotated as response to oxygen-containing compounds, negative regulation of cell death, etc. (*Chi-square* test, *p* < 0.05, *df* = 1, *two-sided*) (Figure 7C).

We identified 1,102 early-miRNA target genes and 1,049 late-miRNA target genes with available annotations of the GO Cellular Component. Compared with the early-miRNA target genes, a significantly greater proportion of the late-miRNA target genes were annotated as enzymes encodingcytoplasmic vesicles, secretory vesicles and phagocytic vesicles (chi-square test, *p* < 0.05, *df* = 1,two-sided), and a significantly lower proportion of the target genes were annotated as intrinsic components of the plasma membrane (*chi-square* test, *p* < 0.05, *df* = 1, *two-sided*) (Figure 7D).

We identified 983 early-miRNA target genes and 930 late-miRNA target genes with available annotations of the GO Cellular component. Compared with the early-miRNA target genes, the late-miRNA targets represented a significantly greater proportion of the target genes annotated as enzymes involved in post-translational protein modification, intra-celluar signalling by second messengers, the cell cycle and myogenesis (*chi-square* test, *p* < 0.05, *df* = 1,*two-sided*) and a significantly lower proportion of the target genes annotated as axon guidance and heme metabolism (*chi-square* test, *p* < 0.05, *df* = 1,*two-sided*) (Figure 7E).

We also investigated the propensity of the target genes of early-miRNAs and late-miRNAs encoding essential genes in cells (Morgens et al., 2016; Cacheiro et al., 2020). A total of 50 essential categories were evaluated, including 15 partially essential terms and 35 completely essential terms, and 631 and 599 essential genes were identified as target genes of early-miRNAs and late-miRNAs, respectively. Overall, there was no significant difference in the proportion of essential genes encoded by the target genes of early-miRNAs and late-miRNAs (*chi-square* test, p > 0.05, *df* = 1, *two-sided*).

### Construction of predictive models for early- and late-miRNAs based on sequence-structure features

Utilizing the RNA-FM language model and support vector machine (SVM) approaches, we established a predictive model that discriminates early- and late-miRNAs purely based on miRNA sequence and structural features (see *Methods*). We trained the predicitve model on 80% of the dataset, and evaluated its performance in an independent validation set (20% of data). The predictive model achieved 75% classification accuracy (Supplementary Figure S9A). We further adopted the predictive probability to score the propensity of miRNAs located in late-replicating domains, and observed late-miRNAs have significant higher scores of than ealry-miRNA (Supplementary Figure S9B). These results confirmed that the information of replication timing domains is inherently encoded in miRNAs’ sequence-structure signatures, and suggested that late-replication specific mutation patterns leave direct imprints on miRNA architecture.

## Discussion

Cell division is one of the most important events in the cell life cycle (Wu and Li, 2016). Orderly and high-fidelity genome replication ensures that daughter cells maintain growth vitality (Frost et al., 2021). Recent studies revealed the DNA replication temporal pattern of the genome and indicated that the early and late replication domains have significant effects on the epigenetic landscape and genomic mutability. As an important factor in posttranscriptional regulation, the occurrence and retention of miRNAs in evolution depend mainly on whether the pre-miRNA can fold into a thermally stable hairpin structure, which is recognized by Ago2 and Dicer to produce mature miRNAs.

The bias toward AT enrichment of late replication domains clearly poses a considerable challenge for late-miRNAs to maintain a stable hairpin structure. Our study demonstrated that miRNAs might take advantage of this mutation bias mechanism to extend at both ends, increasing the length of the stems to compensate for the increase in entropy caused by the increase in the number of loops and the relatively lower binding energy of A:T basepairing. On the basis of the GC profiles of the upstream regions, downstream regions and miRNAs, the CG content of late-miRNAs takes on a protruding shape, whereas that of early-miRNAs takes on a nearly flat shape. These results indicate that miRNAs have undergone negative selection to maintain some necessary G:C pairings, coupled with their ease of extension at both ends, and that both factors jointly ensure the maintenance of the miRNA hairpin structure. For this reason, late-miRNAs are more likely to be retained in evolution, which was confirmed in the comparative genomic analysis of mice and rat; that is, the rat orthologous regions of late-miRNAs have a greater probability of folding into miRNA-like hairpin structures.

Further analysis of the regions surrounding the miRNAs revealed that late-miRNAs tend to be located in regions with relatively few protein genes and have relatively high expression activities in various tissues. We speculate that late-miRNAs may have a relatively independent transcriptional initiation system. The late-miRNAs tend to coordinately regulate the same type of genes; the target genes exclusively regulated by late-miRNAs have shorter UTRs and lower expression variability and are enriched in functional categories such as exocytosis, the cell cycle, and posttranslational protein modification. These results suggest that the location of the early and late replication domains has a certain effect on the miRNA-mediated posttranscriptional regulatory network. Our research results provide new insight into the study of miRNA structural genomics and would help us understand the evolutionary mechanism of miRNAs and miRNA-mediated posttranscriptional regulation in cancers (Wu et al., 2023; Ma et al., 2024).

To explore the generalizability of the observed miRNA features in human genome, we extracted the early- and late-replicating domains in human embryonic stem cells (GSE137764) (Zhao et al., 2020), and obtained 1,372 early-pre-miRNAs and 391 late pre-miRNAs. The sequence analyisis demonstrated the similar tendancy that the A or U enriched in the late-mRNAs (Supplementary Figure S10A,B), however, the simulation of post-regulation network analysis among miRNAs and their experimental target genes (miRTarBase) did not reveal a significant tendency for late mRNAs to co-regulate the same target genes in human genome (data not shown). We propose that the following reasons may potentially explain this inconsistency. First, the population genetics may be different between human and mouse, the mouse species are characterized by high reproductive rates and short lifespans, maintain elevated genetic diversity. In contrast, humans exhibit lower reproductive rates and extended lifespans, leading to slower accumulation of neutral mutations and adaptive variants. The potential higher probability of fixation of environmentally adaptive mutations may explain the observed coordinated regulation patterns of late-miRNAs in mouse genome. Second, there were significant structural divergence in 3′UTR regions between human and mouse, the human 3′UTRs display greater sequence complexity and regulatory element diversity, the increased density of miRNA binding sites in human 3′UTRs may impose additional regulatory constraints, thereby reducing the likelihood of observing conserved co-regulatory patterns. Consequently, the synergistic regulatory effects observed in mouse may stem from their higher fixation rates of environmental adaptive mutations.

## Data Availability

The original contributions presented in the study are included in the article/Supplementary Material, further inquiries can be directed to the corresponding author.

## Supplementary material

SUPPLEMENTARY FIGURE S1 The early-miRNAs and late-miRNAs have similar folding ensemble properties. **(A)** Comparison of the folding free energy of the minimum energy structure between early- and late-pre-miRNAs. **(B)** Comparison of the frequencies of the minimum energy structure in the ensembles between early- and late-pre-miRNAs. **(C)** Comparison of the ensemble diversities between early-and late-pre-miRNAs. The na represents a p value > 0.05.

SUPPLEMENTARY FIGURE S2 The comparison of position-specific nucleotide distributions between early- and late-miRNAs in **(A)** Dicer 5′ motifs and **(B)** 3′ motifs. The p values were calculated in for each base. The * represents a p value≤0.05, ** represents a p value ≤ 0.01, and na represents a p value > 0.05.

SUPPLEMENTARY FIGURE S3 The clustering analysis of Dicer 5′ cleavage motifs. **(A)** The multidimensional scaling (MDS) scatter plot of miRNAs, with early-miRNAs represent red nodes an late-miRNAs as blue nodes. **(B)** The late-miRNAs have a significantly higher value of the first component than early-miRNAs. **(C)** The early-miRNAs have similar value of the second component with late-miRNAs.

SUPPLEMENTARY FIGURE S4 The clustering analysis of Dicer 3′cleavage site motifs. **(A)** The multidimensional scaling (MDS) scatter plot of miRNAs, with early-miRNA represent red nodes an late-miRNAs as blue nodes. **(B)** The late-miRNAs have a significantly higher value of the first component than early-miRNAs. **(C)** The late-miRNAs have a significantly lower value of the first component than early-replicating miRNA.

SUPPLEMENTARY FIGURE S5 The early-miRNAs and late-miRNAs loci have significantly different promoter properties in **(A)** the GC ratios, **(B)** the TATA-box affinity, **(C)** distance between promoters and the pre-miRNAs. * represents a p value≤0.05, ** represents a p value ≤ 0.01.

SUPPLEMENTARY FIGURE S6 Simulation analysis to explore the target-recognition preference of early-miRNA and late-miRNAs. Distribution of the number of genes regulated by both the early-miRNAs and late-miRNAs in 100 simulations. The arrow represents the observed number of targets of both the early-miRNAs and late-miRNAs.

SUPPLEMENTARY FIGURE S7 Simulation analysis to explore cross-domain target-recognition preference of early-miRNA and late-miRNAs. **(A)** Distribution of the number of early-genes regulated by early-miRNAs, and late-genes regulated by late-miRNAs in 100 simulations. The arrow represents the observed number of regulation-ships between early-genes regulated by early-miRNAs, and late-genes regulated by late-miRNAs. **(B)** Distribution of the number of regulation-ships between early-genes regulated by late-miRNAs, and late-genes regulated by early-miRNAs in 100 simulations. The arrow represents the observed number of the early-genes regulated by late-miRNAs, and the late-genes regulated by early-miRNAs.

SUPPLEMENTARY FIGURE S8 The target genes of early-miRNAs and late-miRNAs tend to have short lenght of 3′UTRs and lower transcription variations. **(A)** Comparison of 3′UTR length of targets genes between early-miRNAs and late-miRNAs. **(B)** Comparison of transcriptional variation of target genes between early-miRNAs and late-miRNAs. * represents a p value≤0.05, and ** represents a p value ≤ 0.01.

SUPPLEMENTARY FIGURE S9 The performance of predictive model in classify the early- and late-miRNAs. **(A)** The ROC plot showing the model achieved 75% classification accuracy in the independent validation set. **(B)** Using the predictive probability to represent the propensity of miRNAs located in late-replicating domains, the late-miRNAs have significant higher scores thant ealry-miRNA. * represents a p value≤0.05, ** represents a p value ≤ 0.01.

SUPPLEMENTARY FIGURE S10 The early-miRNAs and late-miRNAs have significantly different sequence properties in human genome. **(A)** Comparison of the mononucleotide composition in the sequences of early- and late-pre-miRNAs. **(B)** Comparison of the dinucleotide compositions of the sequences of early- and late-miRNAs. The * represents a p value≤0.05, ** represents a p value ≤ 0.01, and na represents a p value > 0.05.
