# Supplementary figures and images for: Insights into the evolution and regulation of miRNAs from the view of their DNA replication temporal domains
Source: Front Genet. 2025 Jun 23;16:1544802. doi: 10.3389/fgene.2025.1544802 (PMC12230444; doi:10.3389/fgene.2025.1544802)

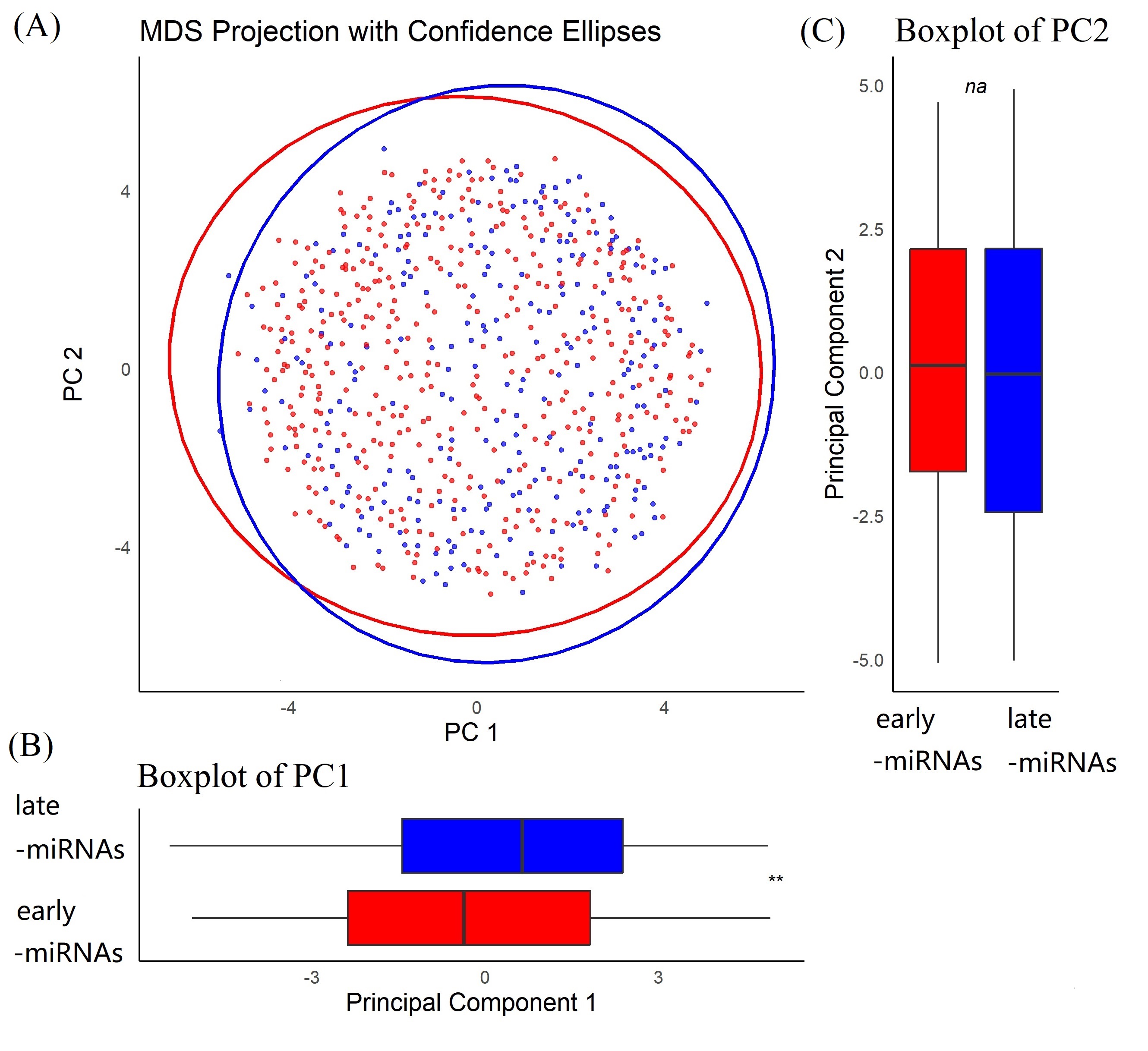

Supplement: Supplementary file 1 [file Image3.jpeg]

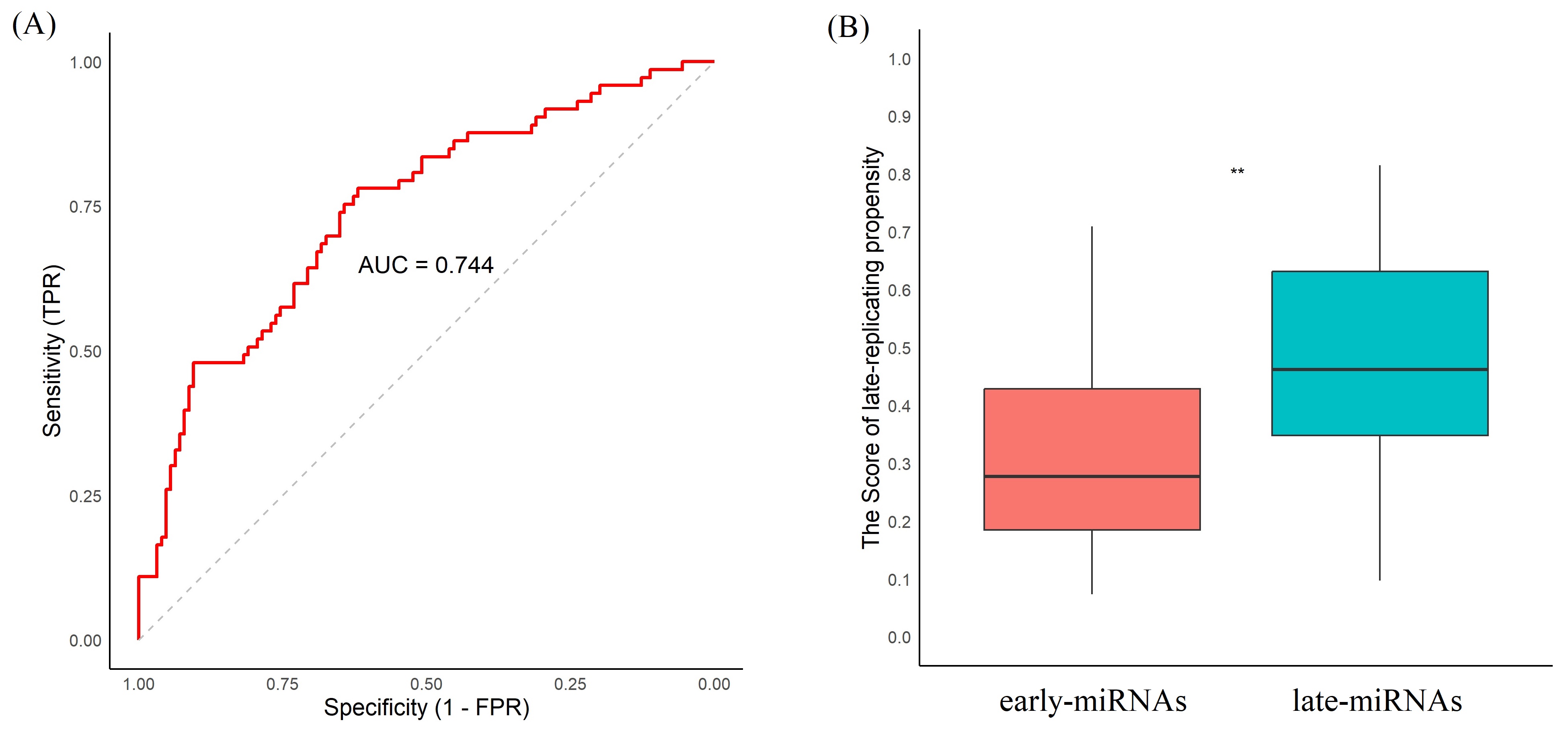

Supplement: Supplementary file 2 [file Image9.jpeg]

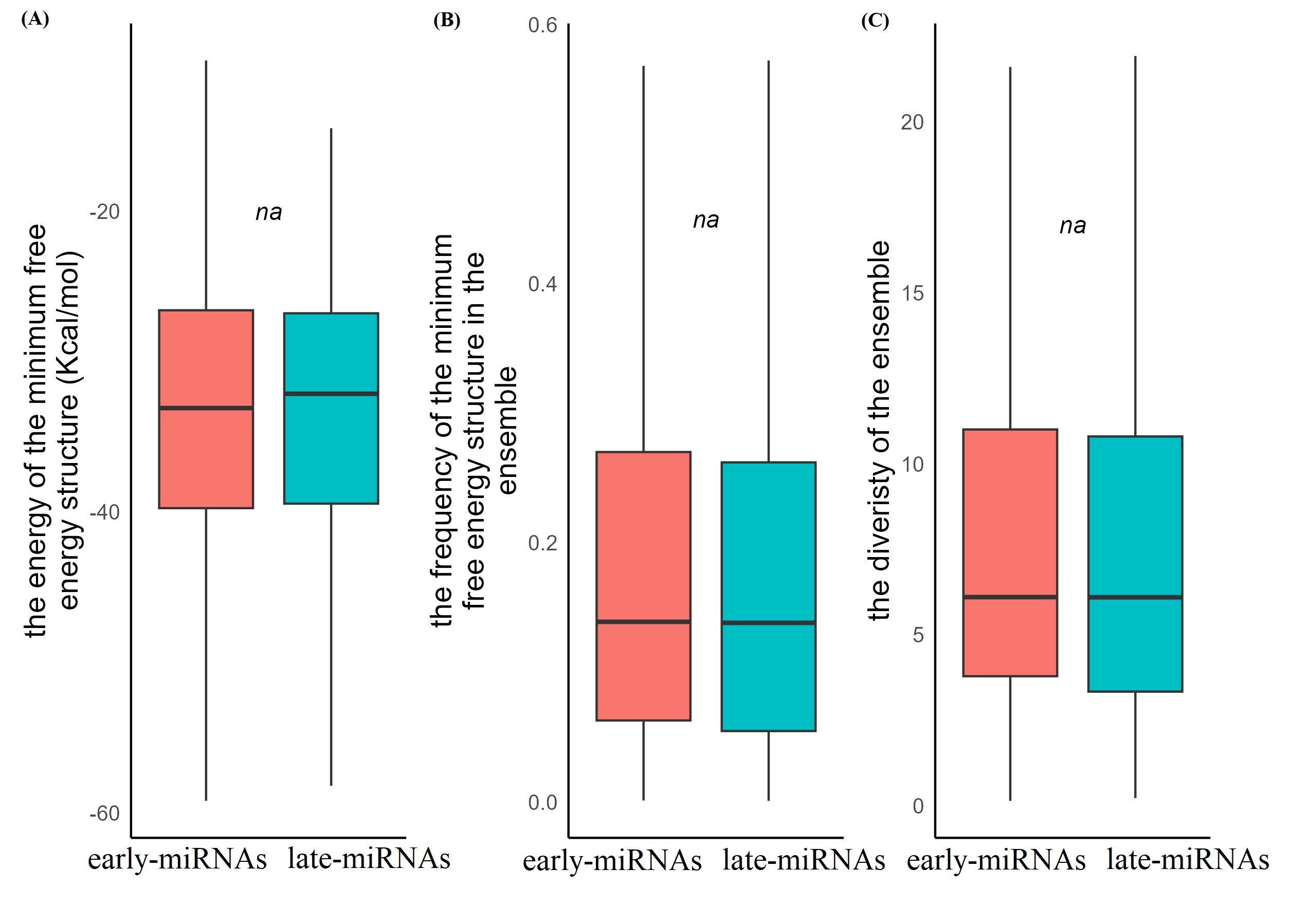

Supplement: Supplementary file 3 [file Image1.jpeg]

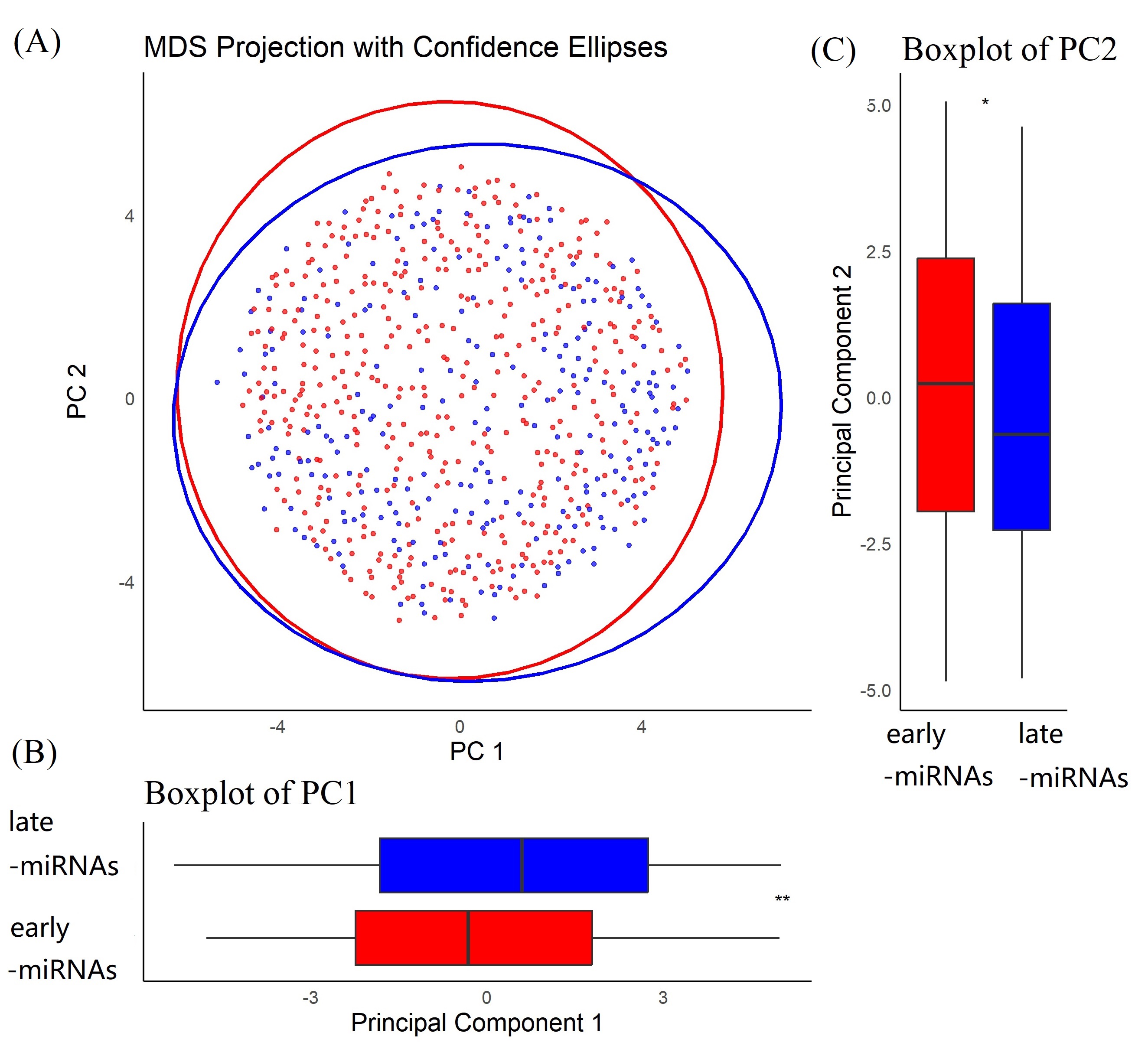

Supplement: Supplementary file 4 [file Image4.jpeg]

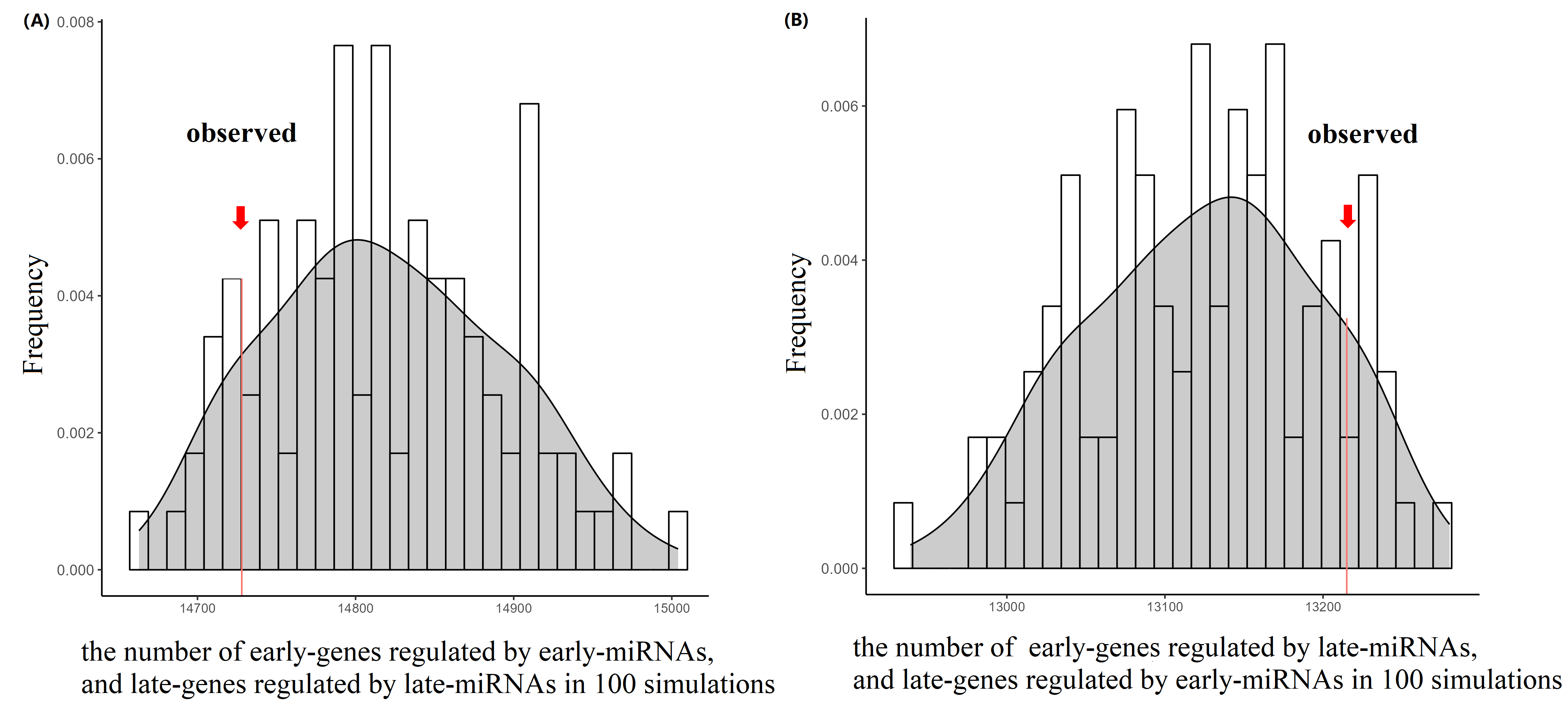

Supplement: Supplementary file 5 [file Image7.jpeg]

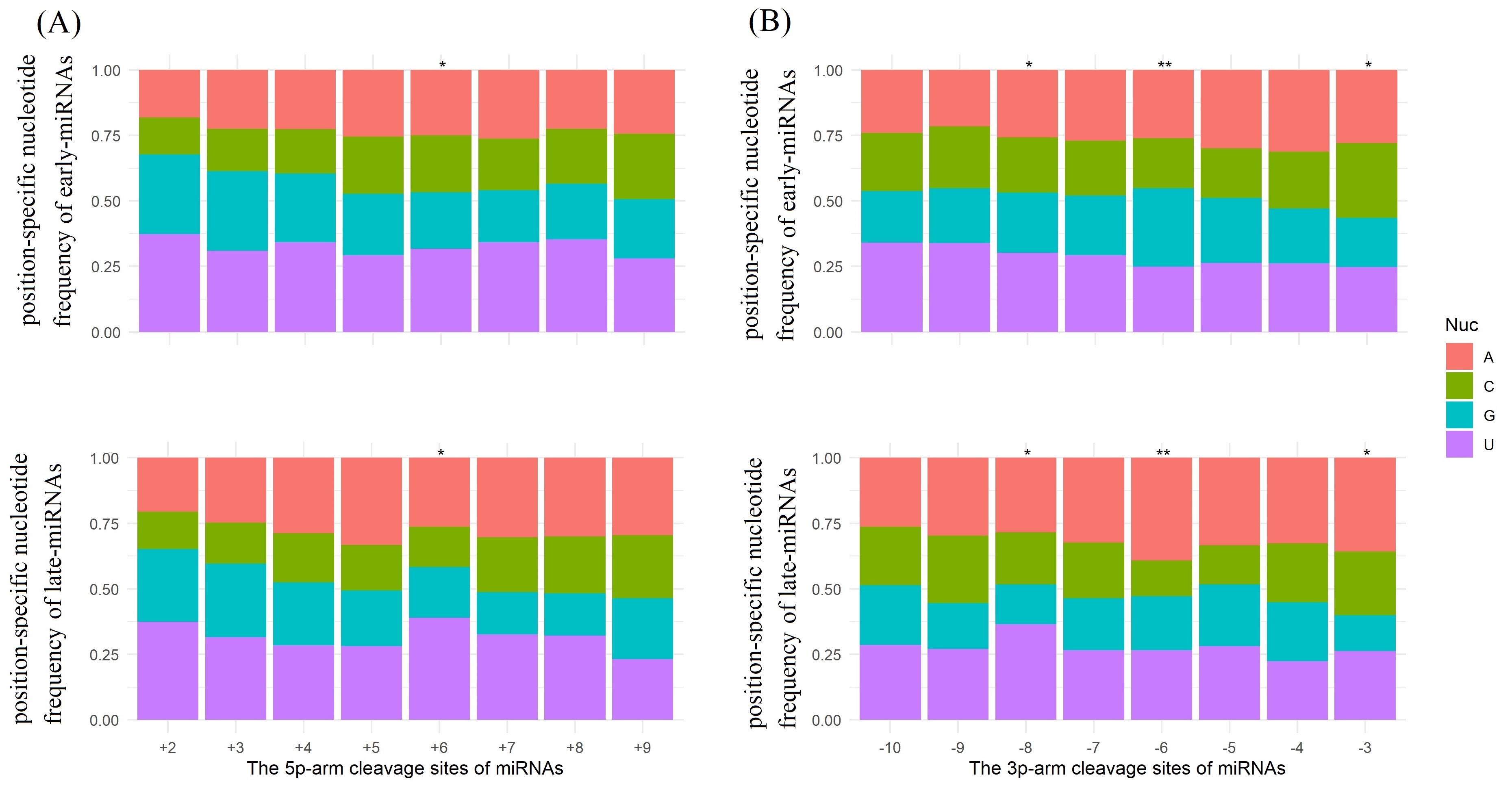

Supplement: Supplementary file 6 [file Image2.jpeg]

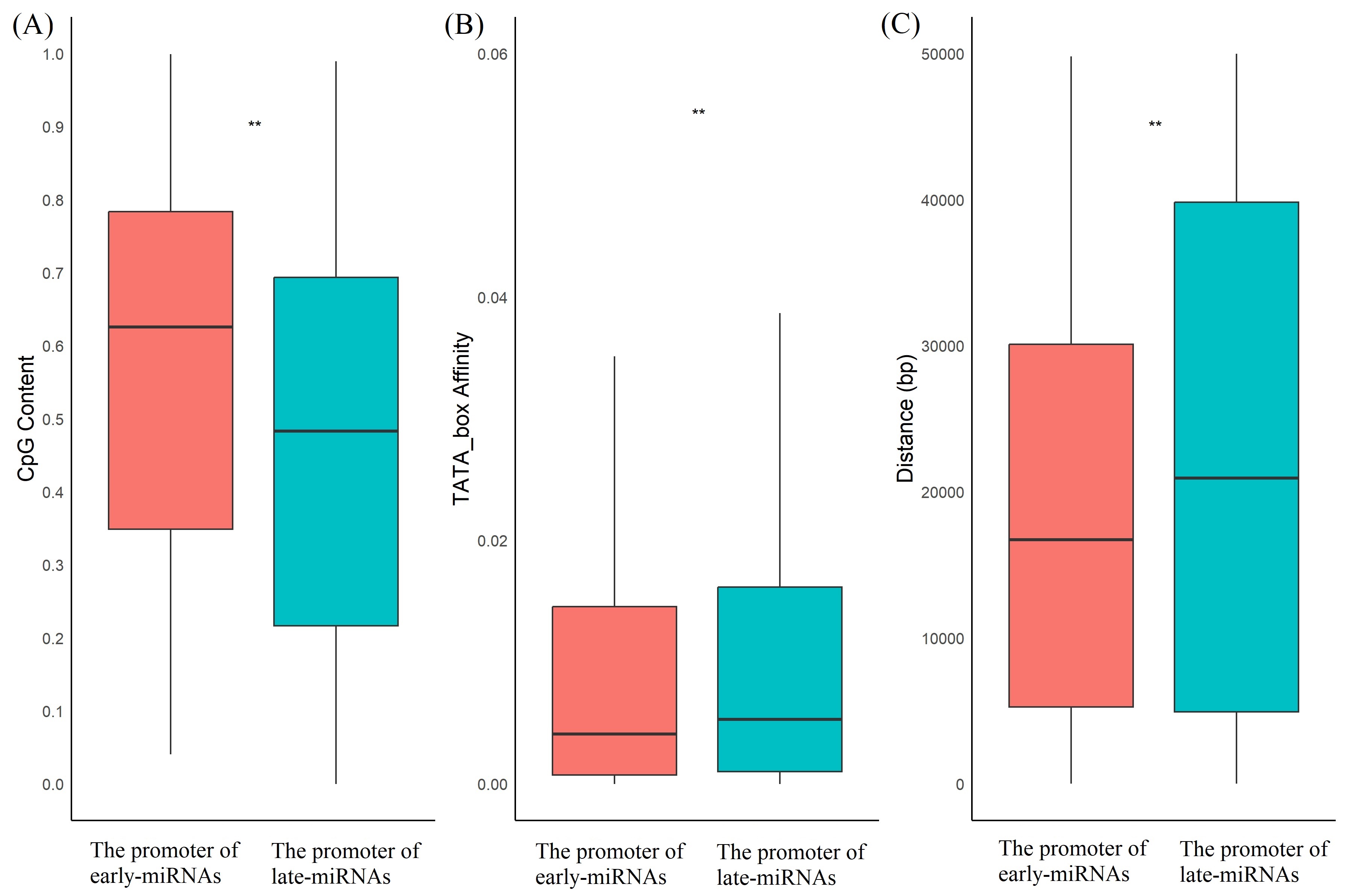

Supplement: Supplementary file 7 [file Image5.jpeg]

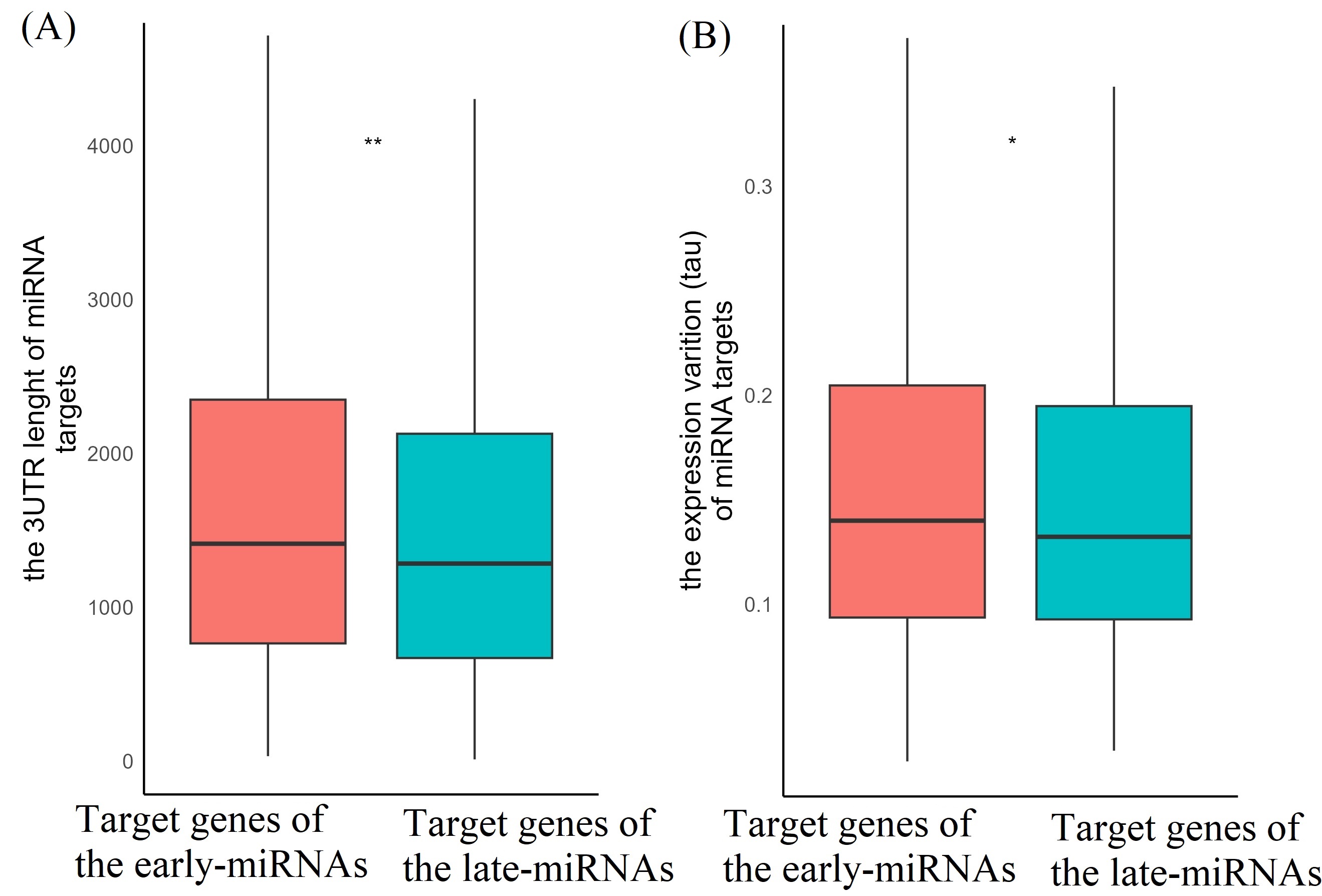

Supplement: Supplementary file 8 [file Image8.jpeg]

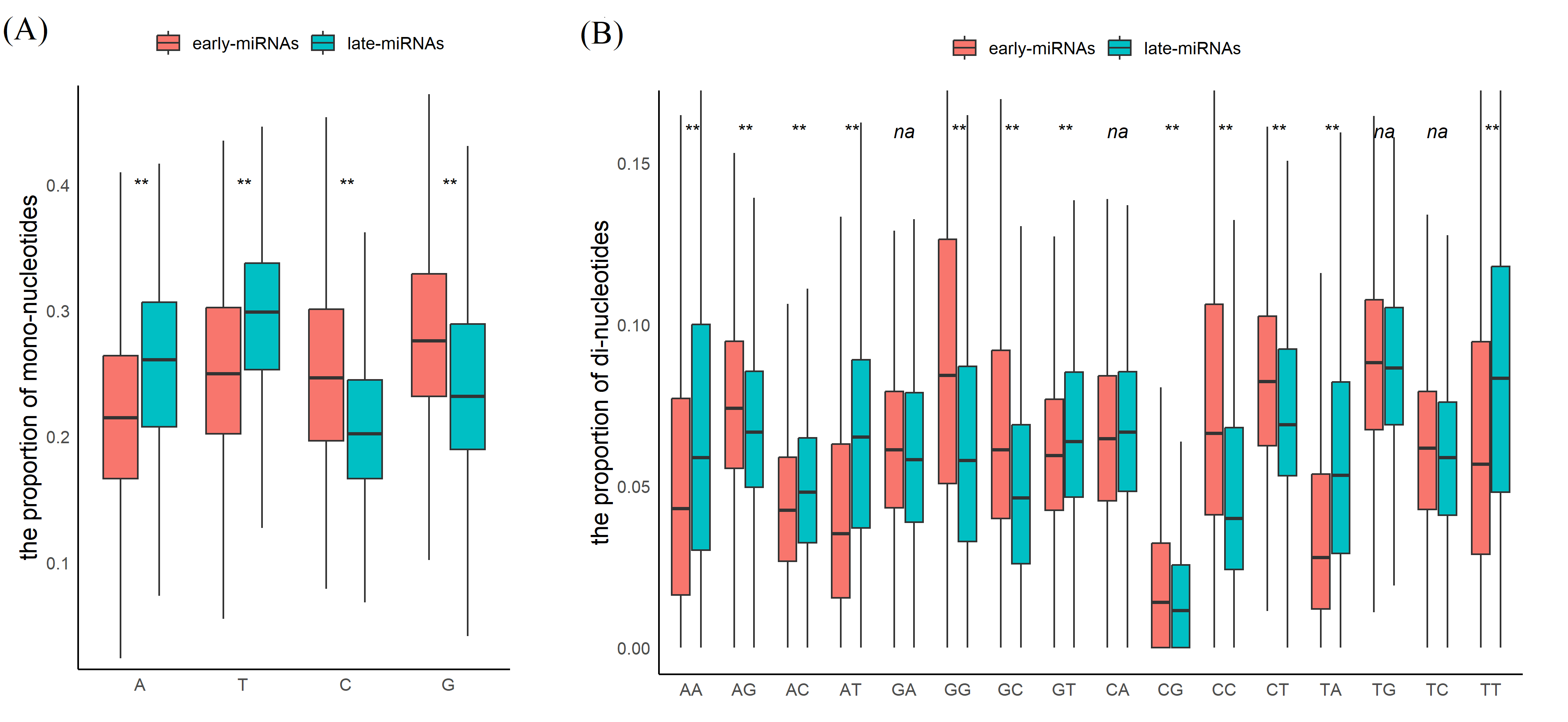

Supplement: Supplementary file 9 [file Image10.png]

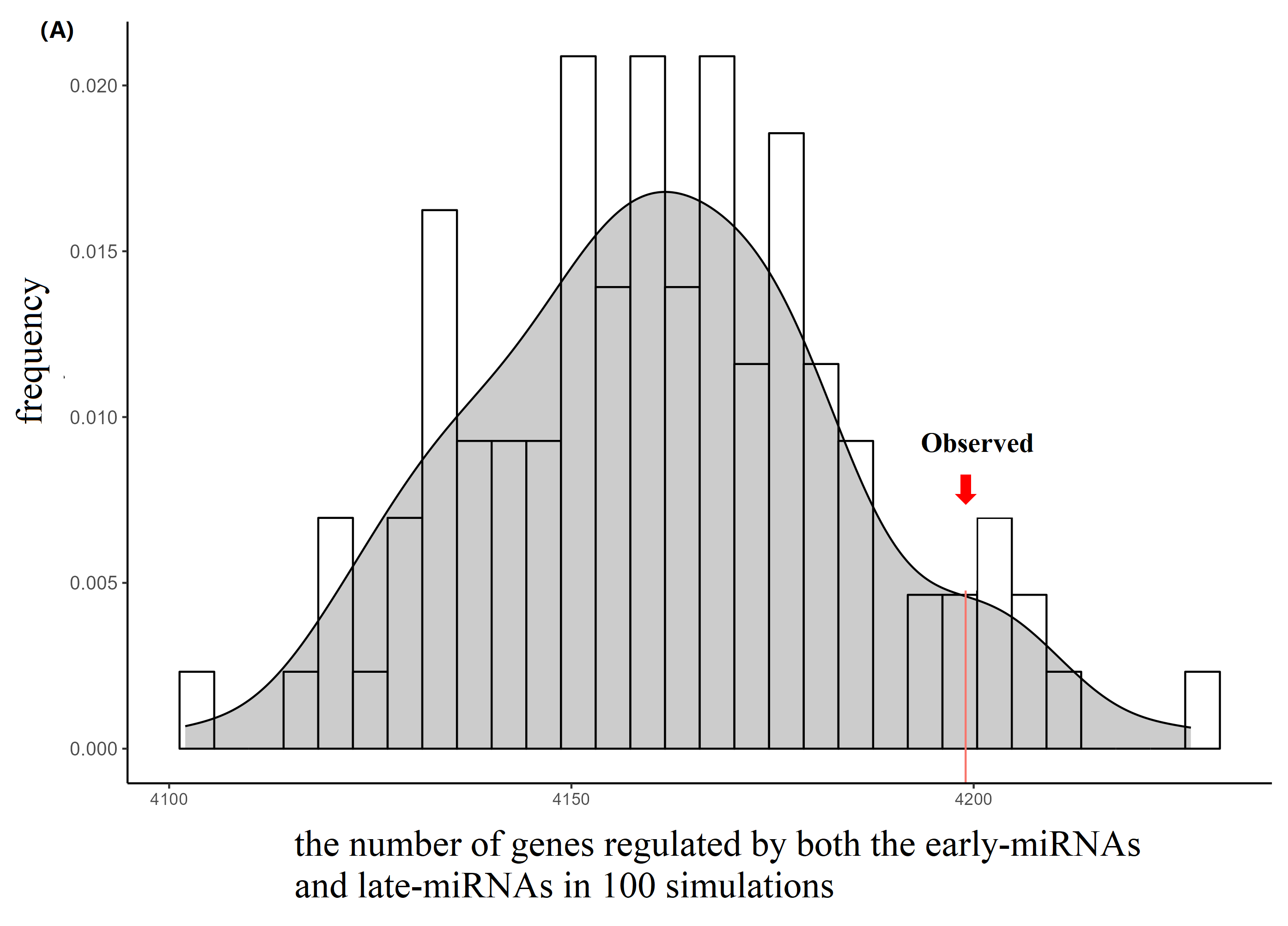

Supplement: Supplementary file 10 [file Image6.jpeg]
